# Mesenchymal Stromal Cells Rapidly Suppress TCR Signaling-Mediated Cytokine Transcription in Activated T Cells Through the ICAM-1/CD43 Interaction

**DOI:** 10.3389/fimmu.2021.609544

**Published:** 2021-02-22

**Authors:** Shuwei Zheng, Ke Huang, Wenjie Xia, Jiahao Shi, Qiuli Liu, Xiaoran Zhang, Gang Li, Jieying Chen, Tao Wang, Xiaoyong Chen, Andy Peng Xiang

**Affiliations:** ^1^Key Laboratory for Stem Cells and Tissue Engineering, Ministry of Education, Center for Stem Cell Biology and Tissue Engineering, Sun Yat-sen University, Guangzhou, China; ^2^Department of Pediatrics, Sun Yat-sen Memorial Hospital, Sun Yat-Sen University, Guangzhou, China; ^3^Guangzhou Blood Centre, Institute of Blood Transfusion, Guangzhou, China; ^4^The Biotherapy Center, The Third Affiliated Hospital, Sun Yat-sen University, Guangzhou, China; ^5^Department of Pathophysiology, Zhongshan School of Medicine, Sun Yat-sen University, Guangzhou, China; ^6^Department of Biochemistry, Zhongshan School of Medicine, Sun Yat-sen University, Guangzhou, China; ^7^Guangzhou Regenerative Medicine and Health Guangdong Laboratory, Guangzhou, China

**Keywords:** mesenchymal stromal cells, T cells, TCR signaling, ICAM-1, CD43

## Abstract

Cell-cell contact participates in the process of mesenchymal stromal cell (MSC)-mediated T cell modulation and thus contributes to MSC-based therapies for various inflammatory diseases, especially T cell-mediated diseases. However, the mechanisms underlying the adhesion interactions between MSCs and T cells are still poorly understood. In this study, we explored the interaction between MSCs and T cells and found that activated T cells could rapidly adhere to MSCs, leading to significant reduction of TNF-α and IFN-γ mRNA expression. Furthermore, TCR-proximal signaling in activated T cells was also dramatically suppressed in the MSC co-culture, resulting in weakened Ca^2+^ signaling. MSCs rapidly suppressed TCR signaling and its downstream signaling in a cell-cell contact-dependent manner, partially through the ICAM-1/CD43 adhesion interaction. Blockade of either ICAM-1 on MSCs or CD43 on T cells significantly reversed this rapid suppression of proinflammatory cytokine expression in T cells. Mechanistically, MSC-derived ICAM-1 likely disrupts CD43-mediated TCR microcluster formation to limit T cell activation. Taken together, our results reveal a fast mechanism of activated T cell inhibition by MSCs, which provides new clues to unravel the MSC-mediated immunoregulatory mechanism for aGVHD and other severe acute T cell-related diseases.

## Introduction

Mesenchymal stromal cells (MSCs) have attracted great interest as a form of cell therapy because of their self-renewal capacity, multipotency, and potent immunomodulatory effects on both innate and adaptive immune cells (1–3). Although numerous preclinical and clinical studies have shown that MSCs can be therapeutically relevant in a variety of inflammatory and autoimmune diseases (4–9), certain obstacles still limit the translation of stem cell therapy into practice. For example, the immune-suppressive properties of MSCs have been successfully shown to control clinical severe graft-versus-host disease (GVHD) and improve survival (8, 9). However, clinical studies have not yet provided conclusive evidence of the efficacy of such strategies, partially due to the heterogeneity in study design (such as patient characteristics and GVHD severity) and in the MSC products used (10–13) (including source, fresh vs. cryopreserved; expansion medium; etc.). Importantly, we lack a comprehensive understanding of the mechanisms underlying the therapeutic activity of MSCs.

MSCs exert their immunosuppressive effects through paracrine factors or via physical contact with inflammatory cells (5, 14). The factors and pathways currently known to be associated with the immunomodulatory actions of MSCs include indoleamine 2,3-dioxygenase (IDO), prostaglandin E2 (PGE2), heme oxygenase-1 (HO-1), transforming growth factor beta (TGF-β), and others (5). Although paracrine mechanisms acting through the secretion of various soluble factors play important roles in the immunomodulatory capacity of MSCs, a number of studies have demonstrated that the immunosuppressive activity of MSCs increases upon their direct contact with activated immune cells (14–16). As most of the relevant soluble factors have a limited range of diffusion, MSCs need to be in close proximity to their target cells to exert their immunosuppressive effect. For example, MSCs incubated with T lymphocytes display significantly higher immunosuppressive activity under contact conditions than under separating conditions (17, 18). Preclinical and clinical data also demonstrate that the therapeutic efficacy of MSCs largely depends on their ability to migrate to injured tissues. For example, localized injection of MSCs in the colon has shown promising outcomes in complex anal fistulas (19, 20), while intravenous injection of MSCs yields unsatisfactory treatment effects in the same disorder (21, 22), potentially indicating that an insufficient number of intravenously injected MSCs homed to the target tissue. Therefore, it is reasonable to assume that a direct cell-cell interaction leads to a more significant impact on MSC activity.

Previous studies have demonstrated that adhesion molecules are involved in the interactions between early T cells and mesenchymal bone marrow stromal cells (23). Ren et al. further elucidated the importance of cell–cell contact in the MSC-mediated immunosuppression of T cells and reported the involvement of intercellular adhesion molecule-1 (ICAM-1) and vascular cell adhesion molecule-1 (VCAM-1), indicating that adhesion molecules play critical roles in MSC-mediated suppression of T cell proliferation (24). Most recently, Silva-Carvalho et al. observed that MSCs primed with GVHD patient plasma exhibited an enhanced immune-suppressive capacity that was correlated with increased expression of ICAM-1 (25). Functional blockade of adhesion molecules has been shown to significantly reverse MSC-mediated T cell suppression (16), but the exact mechanism underlying this suppression is still unknown. Therefore, further exploration of the interaction between MSCs and T cells is needed to clarify the behavior of these cells in the inflammatory environment and to reveal the mechanisms underlying MSC alleviation of aGVHD and other T cell-mediated diseases.

## Methods

### Ethics Approval and Consent to Participate

The study protocol was approved by the Institutional Ethics Committee of Zhongshan School of Medicine of Sun Yat-sen University and was conducted in accordance with the principles of the Declaration of Helsinki. All patients provided written informed consent.

### Isolation and Characterization of MSCs

After obtaining informed consent, human bone marrow samples were collected from healthy donors. MSCs were isolated from bone marrow and cultured as described previously (26). Culture-expanded MSCs exhibited surface expression of CD29, CD44, CD73, CD90, CD105, and CD166 but not CD34 or CD45 (Supplementary Figure 1A). At the sixth passage, the multipotent differentiation capacity of the MSCs was confirmed by their forced differentiation into osteoblasts, chondrocytes, or adipocytes (Supplementary Figure 1B), which was performed as described previously (26).

### Processing of Peripheral Blood Cells and T Cell Sorting

For *in vitro* coculture experiments, peripheral blood was obtained from healthy donors and pediatric patients with steroid refractory acute GVHD (aGVHD). Peripheral blood mononuclear cells (PBMCs) were isolated by centrifugation using Ficoll-Paque^TM^ Plus (1.077 g/mL, GE Healthcare) density gradient centrifugation and were used in the following experiments. For T cell sorting, it was performed according to the guidelines for the use of flow cytometry and cell sorting in immunological studies (30).

### RNA Isolation, Reverse Transcription, and Real-Time qRT-PCR

T cells labeled with CellTracker dyes before coculture were harvested and then rinsed gently using the tip. Next, labeled T cells were sorted directly into TRIzol for qPCR. Total RNA was extracted using TRIzol reagent (Molecular Research Center, Inc.) according to the manufacturer's instructions. Reverse transcription was performed using oligo-dT primers (Thermo), and quantitative real-time qRT-PCR was performed using SYBR PCR Master Mix (Roche) according to the manufacturer's instructions. qRT-PCR was conducted in triplicate for each sample, and three independent experiments were performed. Signals were detected using a Light Cycler 480 detection system (Roche). The primer sequences are listed in Supplementary Table 1.

### Western Blotting

T cells labeled with CellTracker dyes (Thermo Fisher) before coculture were harvested and then fixed with 10% buffered formalin phosphate (10% formalin) (27). The labeled T cells were sorted for immunoblotting. Jurkat and primary T cells were washed with PBS and lysed on ice in PhosphorSafe^TM^ Extraction Reagent (EMD Millipore Corporation). Then, the proteins were separated by SDS-PAGE and transferred to a polyvinylidene fluoride (PVDF) membrane (Immobilon-P; Millipore) using a semidry transfer system (Integrated Separation Systems). After being blocked for 1 h in 5% BSA, the membranes were incubated with a primary antibody for 12 h at 4°C and were then washed and incubated for 1 h with an appropriate HRP-conjugated secondary antibody. Antigen-antibody complexes were detected via enhanced chemiluminescence (GE Healthcare). All the antibodies used are listed in Supplementary Tables 2, 3. All the immunoblotting experiments were performed at least three times, and representative data are shown.

### T Cell Stimulation and Coculture

Cells were stimulated with soluble antibodies against CD3 (10 μg/mL) plus CD28 (10 μg/mL) for 60 min at 37°C and were then cultured with MSCs for 60 min, while T cells from pediatric aGVHD patients were cocultured directly with MSCs. PBMC T/Jurkat cells and MSCs were plated in a 5:1 ratio. The T cells were then isolated and used for subsequent experiments, such as western blotting analysis, mRNA analyses, phospho-protein staining for flow cytometry, immunofluorescence, and Ca^2+^ analysis. For the CD43-blocking experiments, T cells were exposed to an anti-CD43 mAb (10 μg/mL; Thermo Fisher) before coculture (28). For the ICAM-1-blocking experiments, MSCs were exposed to 10 μg/mL anti-ICAM-1 mAb (R&D Systems) before coculture (29). For other stimuli, the same procedure was performed as for anti-CD3/28 mAbs.

### Ca^2+^ Loading and Analysis in T Cells

T cells were labeled with the calcium indicator Fluo-4 AM (Thermo Fisher). A total of 10^6^ cells in 1 mL of RPMI 1640 medium containing 10% FBS were loaded with 5 mM Fluo-4 AM for 20 min at 37°C in the presence of 0.1% Pluronic F-127 and 0.25 mM Probenecid (Thermo Fisher). The cells were washed three times with RPMI 1640 and incubated at 37°C for an additional 5 min. The cells were then resuspended in RPMI 1640 medium containing 10% FBS, and their Ca^2+^ levels were assessed using a CytoFLEX Flow Cytometer (Beckman Coulter). For live-cell microscopy, loaded T cells were plated on poly-L-lysine-coated culture plates, centrifuged at 100 g for 2 min, and placed in an incubator at 37°C for 30 min. Images were acquired using a BioTek-lionheart FX (BioTek).

### Phospho-Epitope Staining for Flow Cytometry

Treated and untreated T cells were harvested, washed twice with PBS, resuspended in 100 μL PBS, and then fixed and permeabilized using an intracellular fixation and permeabilization buffer set (Thermo Fisher). Briefly, the cells were incubated in fixative for 30 min at RT in the dark, pelleted, washed twice with 2 mL 1 × permeabilization buffer, and then resuspended in 1 × permeabilization buffer at 0.5–1 × 10^6^ cells per 100 μL. The optimal concentrations of fluorophore-specific Abs were added, and the mixtures were incubated for 30 min at RT in the dark. In experiments using a primary antibody and conjugated secondary antibody, the fluorochrome-labeled secondary antibody was optimally diluted (per the manufacturer's instructions) in 3% BSA/PBS, and the cells were resuspended in the same solution. The cells were incubated for at least 30 min at RT or 4°C in the dark, washed with 2 mL 1 × permeabilization buffer, and resuspended in 300 μL staining medium. Flow cytometry was performed on a CytoFLEX instrument (Beckman Coulter), and data were analyzed with FlowJo software (Becton Dickinson). All of the utilized antibodies are listed in Supplementary Tables 4, 5.

### ELISA

The levels of TNF-α and IFN-γ in the supernatants were detected using commercially available ELISA kits (all from R&D Systems) according to the manufacturer's recommended procedures.

### Immunofluorescence

T cells were plated onto poly-L-lysine-coated glass slides placed on the plate, centrifuged at 100 g for 2 min, and placed in an incubator for 30 min to allow the T cells to adhere. The supernatant was removed from each well, and the cells were blocked for 30 min with PBS-Tween 0.05% plus 5% goat serum. The cells were then incubated with the appropriate primary and secondary antibodies overnight at 4°C and for 30 min at room temperature. Nuclei were visualized with DAPI (Fluka). Images were acquired under an LSM 880 with Airyscan (Zeiss). The utilized primary and secondary antibodies are listed in Supplementary Tables 6, 7.

### Cell Line and siRNA Transfection

The Jurkat E6.1 T-cell line was purchased from ATCC. Jurkat T cells (2 × 10^6^/mL) were transfected with CD43-specific siRNA, while MSCs were transfected with ICAM-1–specific siRNA (RiboBio). Transfections were performed using Lipofectamine® RNAiMAX Reagent (13778-150; Thermo Fisher). Cells were cultured for 48 h and analyzed by PCR to determine the interference efficiency.

### Transwell Assays

A Transwell chamber system with 0.4 μm-pore membrane filters (Millipore) was used. Each upper chamber was loaded with T cells (5 × 10^5^ /well) and each lower chamber was loaded with MSCs (1 × 10^5^/well), or vice versa. The chambers were incubated for 1 h at 37°C in a 5% CO_2_ incubator, and the cells remaining on the upper surfaces were removed for subsequent experiments.

### Statistical and Image Analyses

Statistical analysis was carried out using GraphPad Prism version 8.0 software. Images were processed with Imaris (Bitplane). All data are reported as the mean ± SEM of at least three independent experiments. The sample sizes are indicated in the figure legends. Comparisons were performed using a two-tailed Student's *t*-test or two-way ANOVA (for multigroup comparisons). *P*-values less than 0.05 were considered significant, and the level of significance is indicated as follows: ^*^P < 0.05, ^**^P < 0.01, and ^***^P < 0.001.

## Results

### MSCs Rapidly Suppress the Transcription of Cytokines in Activated T Cells

**Figure 1:**
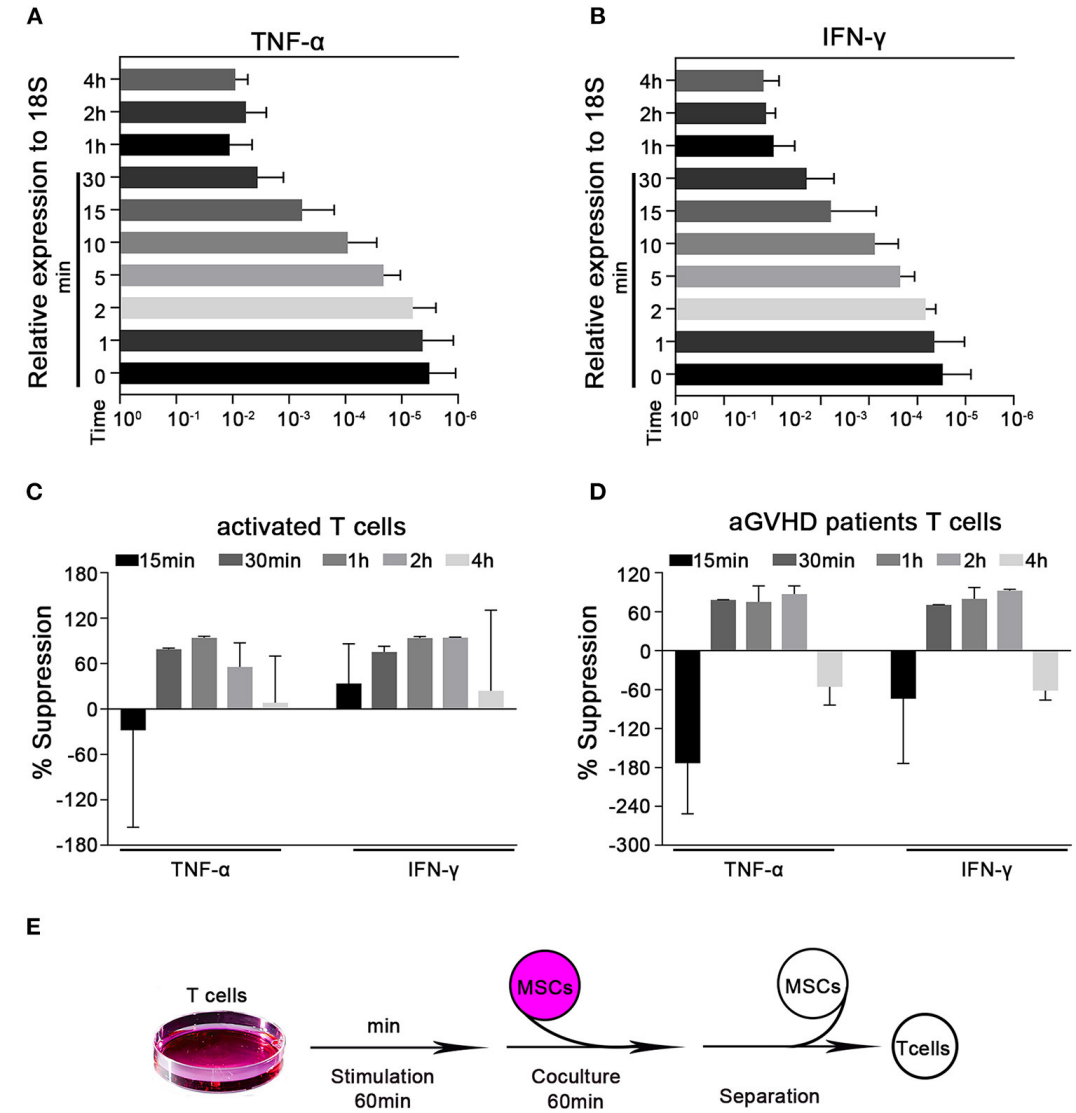
MSCs rapidly suppress the transcription of cytokines in activated T cells. Quantitative reverse transcription–polymerase chain reaction (qRT-PCR) analysis of the mRNA abundances of TNF-α and IFN-γ in activated T cells. **(A,B)** T cells were stimulated by CD3 mAb and CD28 mAb for 1, 2, 5, 10, 15, and 30 min, 1, 2, and 4 h. The mRNA abundances of TNF-α and IFN-γ were analyzed, the results are presented relative to the level of the 18S rRNA. **(C)** Activated T cells from healthy donors were cocultured with MSCs (ratio 5:1) for 15 and 30 min, 1, 2, and 4 h. The T cells were then isolated and analyzed for the mRNA levels of the indicated cytokines, which are shown as the percentages of suppression relative to the corresponding levels in monocultured T cells. **(D)** T cells from pediatric aGVHD patients were treated as described in **(C)**, but without activation. The results are shown as the percentages of suppression by MSCs for the indicated cytokines. **(E)** Schematic of the experimental protocol: T cells were stimulated for 60 min, cocultured with MSCs for 60 min (ratio 5:1), and then isolated for subsequent experiments. All experiments were repeated three times. Data are expressed as the means ± SEM from three donors **(A–C)** or four patients **(D)**.

Cytokine production is a major feature of activated T cells. Such cells have been previously reported to rapidly adhere to MSCs within 4 h and transmigrate into them *in vitro* (31). Here, we established an *in vitro* MSC/activated T cell coculture system and found that activated T cells could adhere to MSCs within 1 h (Supplementary Figure 2). We first used the CD3 mAb and CD28 mAb to activate T cells isolated from the peripheral blood of healthy human donors, and then examined the cytokine protein levels. We found that TNF-α and IFN-γ secretions gradual increased after activation from 30 min to 3 h (Supplementary Figure 3). Considering that this interaction occurs within a short timeframe, we focused on the potential ability of T cells to alter proinflammatory cytokine expression at the mRNA level. We examined the levels of the mRNA transcripts of TNF-α and IFN-γ (the major T cell proinflammatory cytokines) at 1, 2, 5, 10, 15, and 30 min, 1, 2, and 4 h post-activation. We found that the mRNA levels of TNF-α and IFN-γ in preactivated T cells increased in a time-dependent manner within the first hour of activation and appeared to peak at the 1 h timepoint (Figures 1A,B). We then cocultured activated T cells with or without MSCs for 15 and 30 min, 1, 2, and 4 h. As expected, MSCs suppressed the rapid upregulation of the TNF-α and IFN-γ mRNA compared to the levels found in monocultured T cells; this inhibition appeared to peak at the 1 h timepoint (Figure 1C). In addition, cytokine alterations were also evaluated by flow cytometry and ELISA, and the results showed a trend toward MSC-mediated inhibition that was not significant at 1 h; however, the levels of TNF-α and IFN-γ were significantly reduced by MSCs after 2 h (Supplementary Figures 4A–C). Altogether, the mRNA levels could reflect this MSC-mediated rapid modulation in a timely manner. Similar MSC-mediated proinflammatory cytokine inhibition was observed in Jurkat cells, an immortalized cell line of human T lymphocytes (Supplementary Figure 5A). Acute graft-versus-host disease (aGVHD) is a major life-threatening complication initiated by multiple signals that cause alloreactive T-cell activation (32, 33), and MSCs have been successfully used in the treatment of steroid-resistant aGVHD (34–36). To investigate whether the regulatory pathway we were studying contributes to the ability of MSCs to treat aGVHD, we directly cocultured T cells obtained from patients with aGVHD [in whom T cells are activated to different degrees (32)] with MSCs for 15 and 30 min, 1, 2, and 4 h. Consistent with the above-described results, MSCs could reduce the levels of TNF-α and IFN-γ mRNA in T cells derived from aGVHD patients (Figure 1D), with a higher degree of inhibition from 30 min to 2 h. We then selected 1 h as the coculture timepoint for the follow-up experiment presented in Figure 1E. Taken together, our data indicate that MSCs possess the capacity for rapid immunoregulation and may quickly reduce T cell production of proinflammatory cytokines.

### Ca^2+^ Signals in Activated T Cells Are Rapidly Abrogated by MSCs

**Figure 2:**
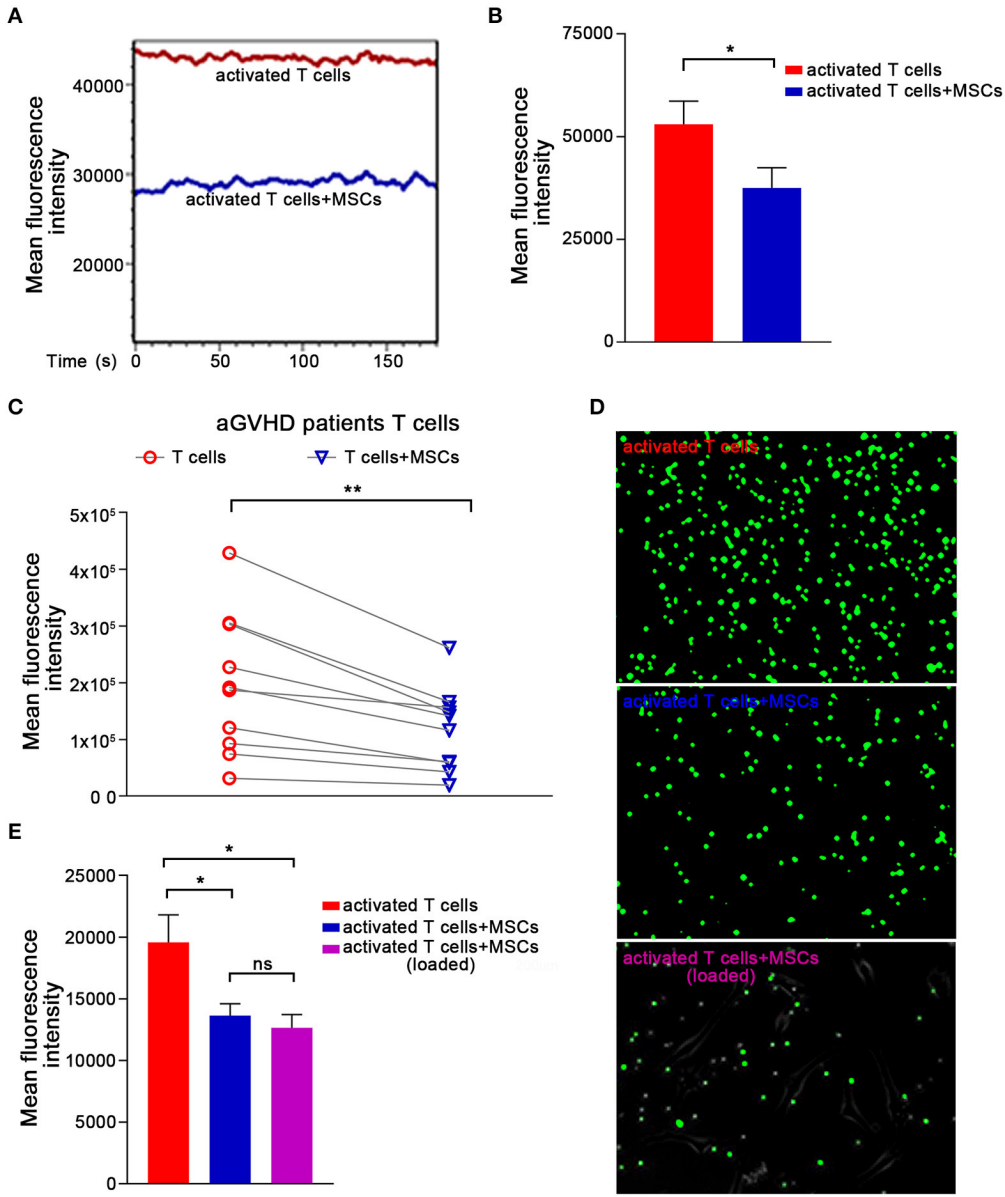
The Ca^2+^ signaling in activated T cells are rapidly abrogated by MSCs. **(A)** T cells were stimulated with CD3mAb and CD28mAb for 1 h, labeled with the Ca^2+^-sensitive dye Fluo-4 AM, and then cocultured with MSCs for 1 h. Activated T cells were analyzed via flow cytometry to monitor dynamic changes in cytosolic Ca^2+^ levels. Data are representative of 10 donors. **(B)** Column chart shows the mean fluorescence intensity (MFI) of the activated T cells described in **(A)**. Data are presented as the means ± SEM from 10 donors. **(C)** T cells from patients were labeled and cocultured as described in **(A)** and assessed by flow cytometry. The data represented were from 10 patients. **(D)** Plates were pre-coated with poly-lysine, loaded (or not) with MSCs, and Ca^2+^ indicator-loaded activated T cells were added and allowed to adhere. T cells (top), T cells removed from the coculture (middle), or those that adhered to the plated MSCs (lowest) are shown. Representative photos were taken by under a live-cell microscope. **(E)** Column displaying the mean fluorescence intensity (MFI) of the activated T cells and MSCs-cocultured activated T cells described in **(D)**. Data are presented as the means ± SEM from three donors. Significant differences are indicated as follows: ns, no statistical significance; *p < 0.05, and **p < 0.01.

Ca^2+^ signaling has been shown to be essential for T-cell proinflammatory cytokine production (37–39). Cyclosporin A and FK506, two immunosuppressants commonly used to treat aGVHD (40–42), reportedly block Ca^2+^ signaling and thus can decrease T lymphocyte cytokine production. Therefore, we further investigated whether MSCs downregulated inflammatory cytokines in preactivated T cells by modulating Ca^2+^ signaling. Preactivated T cells from healthy donors were loaded with the Ca^2+^-sensitive dye Fluo-4 AM, and flow cytometry was used to monitor dynamic changes in Ca^2+^ levels. We found a high Ca^2+^ level in preactivated T cells that were cultured alone and a significantly lower level of Ca^2+^ in preactivated T cells cocultured with MSCs (Figures 2A,B). We also investigated the MSC-mediated Ca^2+^ decreases in T cells that were directly derived from aGVHD patients. As expected, T cells from almost all of the enrolled patients exhibited an obvious and rapid reduction in T-cell Ca^2+^ levels when cocultured with MSCs (Figure 2C). To examine the alteration of Ca^2+^ levels more intuitively, we performed calcium imaging of T cells using Fluo-4 AM. As shown in Figure 2D, the MSC-cocultured group appeared darker than the control activated T cells, regardless of whether the T cells were removed from the MSC plate or left in culture with the MSCs. Mean fluorescence intensity (MFI) analysis also revealed that MSCs could reduce the Ca^2+^ level of activated T cells (Figure 2E). When we used the same protocol to assess the calcium level of Jurkat cells, we found that MSCs rapidly reduced the Ca^2+^ levels in activated Jurkat cells (Supplementary Figures 5B,C). We also analyzed the major calcium-related downstream signaling pathways in activated T cells. As shown in Supplementary Figure 6, the NFAT, NF-κB, and ERK signaling pathways were activated in preactivated T cells, and these pathways were suppressed in preactivated T cells cocultured with MSCs. Taken together, our results indicate that MSCs regulate Ca^2+^ signaling during the MSC-mediated rapid suppression of activated T cells.

### TCR-Proximal Signaling Is Rapidly Altered in Activated T Cells Cocultured With MSCs *in vitro*

**Figure 3:**
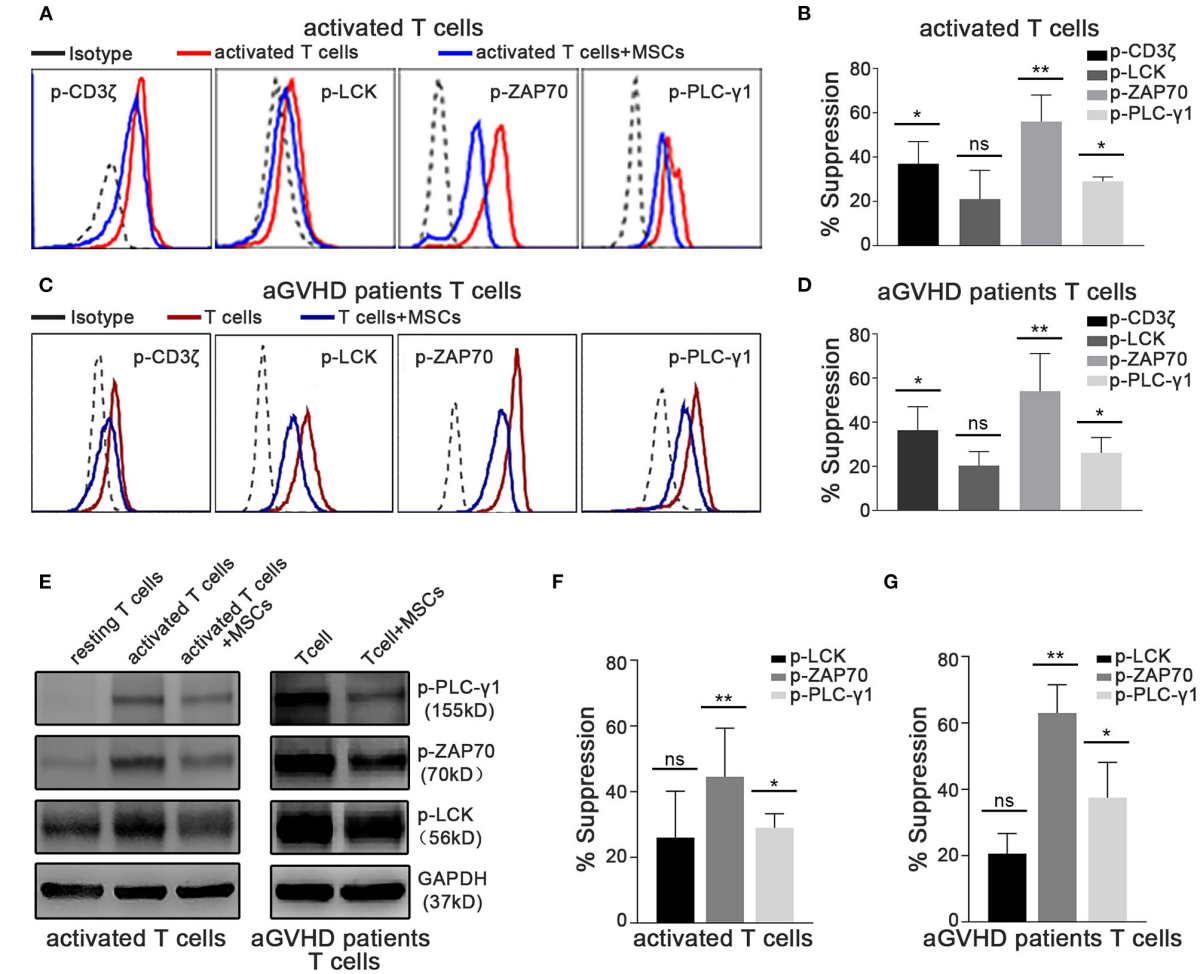
TCR-proximal signaling is altered rapidly in activated T cells cocultured with MSCs *in vitro*. The phosphorylation levels of CD3ζ, LCK, ZAP70, and PLC-γ1 were analyzed. Cocultures were established as described for Figure 1E. **(A)** Flow cytometric analysis of the phosphorylation levels of CD3ζ, LCK, ZAP70, and PLC-γ1 in cocultured and control activated T cells from healthy donors. **(B)** Column chart shows the suppression percentages of the phosphorylation levels by MSCs shown in **(A)**. Data are presented as the means ± SEM from six donors. **(C)** Flow cytometric analysis of the phosphorylation levels of CD3ζ, LCK, ZAP70, and PLC-γ1 in cocultured and control T cells from aGVHD patients. **(D)** Suppression percentages of p-CD3ζ, p-LCK, p-ZAP70, and p-PLC-γ1 in T cells from patients after MSCs coculture. The data are presented as the means ± SEM from six patients. **(E)** Total cell lysates of T cells both from donors and patients were analyzed via western blotting of the listed phosphorylated proteins, with GAPDH detected as a loading control. A representative blot is shown. **(F,G)** Gray-scale values of blots in **(E)** were analyzed, the suppression percentages of listed phosphorylated proteins by MSCs are shown. Data are presented as the means ± SEM from three donors. Significant differences are indicated as follows: ns, no statistical significance; *p < 0.05 and **p < 0.01.

In T lymphocytes, crosslinking of the TCR/CD3 complex, which typically activates cytosolic tyrosine kinases and adaptors, leads to the activation of phospholipase Cγ1 (PLC-γ1), which mediates the upregulation of cytosolic Ca^2+^ levels (43–45). Thus, we further investigated whether MSC-mediated rapid suppression of cytokine transcription occurs through the TCR-Ca^2+^ signaling pathway. Using phosphoflow assays, we found that the level of phosphorylation of CD3ζ, ZAP70, and LCK was significantly increased in activated T cells (Figures 3A,B), as was that of PLC-γ1 (Figures 3A,B), which is a downstream mediator that initiates Ca^2+^ signaling. Importantly, the phosphorylation of CD3ζ, ZAP70, and PLC-γ1 dramatically decreased in activated T cells cocultured with MSCs (Figures 3A,B), suggesting that MSCs can modulate TCR signaling. Moreover, this MSC-mediated downregulation of TCR signaling was also observed in T cells from aGVHD patients (Figures 3C,D). Via western blotting, we found increased levels of phosphorylation of ZAP70, LCK, and PLC-γ1 in activated T cells and T cells from aGVHD patients, as well as suppression of these changes in MSC coculture (Figures 3E–G). Similar results obtained in Jurkat cells (Supplementary Figures 5D,E) further verified that MSC coculture was associated with an obvious blockage of the TCR signaling pathway. As Rasmusson et al. demonstrated that MSC-mediated suppression is dependent on the method used to stimulate lymphocytes (46), we further investigated this MSC-mediated rapid effect on IL-2- or mitogen-driven T cell activation. As shown in Supplementary Figure 7, we found that MSCs do not rapidly modulate IL-2- or mitogen-activated T cells, which further confirms that MSC-mediated rapid effects on T cells are mainly dependent on TCR engagement, as mitogens do not stimulate T cells through the TCR. Our results showed that the TCR-proximal signaling machinery may contribute to MSC-mediated rapid suppression of cytokine transcription in activated T cells.

### MSC-Mediated Rapid Suppression of Activated T Cell Cytokine Transcription Depends on ICAM1-Mediated Cell-to-Cell Contact

**Figure 4:**
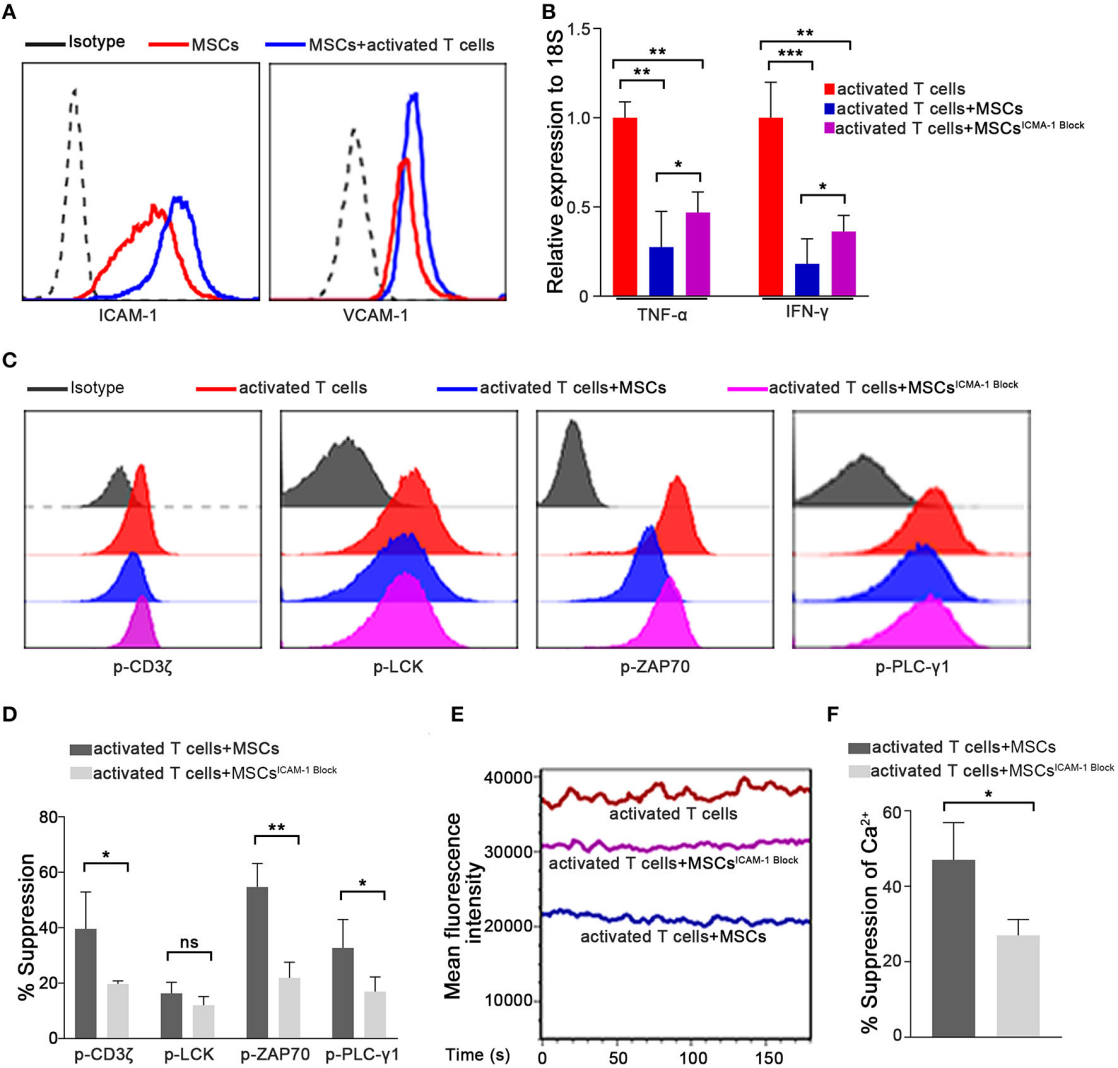
The ability of MSCs to rapidly suppress activated T cell cytokine transcription is dependent on ICAM1-mediated cell-to-cell contact. **(A)** Expressions of ICAM-1 and VCAM-1 on the membranes of MSCs determined by flow cytometry are shown. All experiments were repeated at least three times using the sixth passage MSCs. **(B)** The mRNAs abundances of TNF-α and IFN-γ in activated T cells. Data are shown for activated T cells, activated T cells cocultured with MSCs, and activated T cells cocultured with ICAM-1-blocked MSCs. **(C)** Phosphorylation levels of CD3ζ, LCK, ZAP70, and PLC-γ1 in activated T cells from the three groups described above were examined by flow cytometry. The results presented are representative of those obtained from three independent experiments. **(D)** Bar graphs show the suppression percentages of p-CD3ζ, p-LCK, p-ZAP70, and p-PLC-γ1. Data are shown for activated T cells cocultured with MSCs and activated T cells cocultured with ICAM-1-blocked MSCs. All experiments were repeated three times. **(E)** Cytosolic Ca^2+^ levels were tested by flow cytometry. The mean fluorescence intensity (MFI) is shown. The experimental groups are the same as described in **(B)**. **(F)** Suppression percentages of the Ca^2+^ level in activated T cells from the coculture groups. The results are representative of three independent experiments. Data are presented as the mean ± SEM for each group. Significant differences are indicated as follows: ns, no statistical significance; *p < 0.05, **p < 0.01, and ***p < 0.001.

When the Ca^2+^ levels in T cells from the T/MSC coculture group were measured via live-cell microscopy, we found that the fluorescence intensity was extremely low in MSC-adhered T lymphocytes and progressively increased with the distance from cocultured MSCs (Supplementary Figure 8). This result suggested that direct cell-to-cell communication might play an important role in the MSC-mediated regulation of the Ca^2+^ levels in T cells. To further clarify the underlying mechanism, we used Transwell experiments to investigate whether cell-to-cell contact was essential for the rapid immunosuppressive activity of MSCs in our system. As shown in Supplementary Figure 9, compared to the direct-contact group, MSCs had a much weaker inhibitory effect on the Ca^2+^ levels in the Transwell group. This result was consistent with the changes observed in TCR signaling and the TNF-α and IFN-γ mRNA levels. These observations suggested that cell-to-cell contact plays a critical role in the rapid immunosuppressive activity of MSCs. MSC-mediated immunosuppression has been variably demonstrated to involve many molecules, some of which can be induced by cytokines secreted by activated T cells (5, 14, 16, 47, 48). Next, we further assessed molecules that are potentially associated with the immunomodulatory properties of MSCs, especially those reported to mediate cell-to-cell contact effects, in MSCs cocultured with preactivated T cells for 1 h (Supplementary Figure 10). Of the adhesion molecules thought to be related to MSC-mediated immunosuppression (16), we found that the expression of ICAM-1, which is the most highly expressed adhesion molecule on MSCs, was increased significantly in cocultured MSCs (Figure 4A). ICAM-1-deficient MSCs were recently reported to have reduced immune-suppressive effects *in vitro* and *in vivo* (49, 50). Thus, we speculated that ICAM-1 might contribute to the rapid MSC-mediated regulation of activated T cells. We pretreated MSCs with a blocking antibody against ICAM-1 and then cocultured them with activated T cells, as described above. As expected, ICAM-1 blockade abrogated the MSC-mediated rapid downregulation of TNF-α and IFN-γ in activated T cells compared to that resulting from coculture with control MSCs (Figure 4B). Furthermore, MSC-mediated suppression of TCR signaling (Figures 4C,D) and Ca^2+^ levels (Figures 4E,F) were also partially reversed by ICAM-1 blockade. To confirm the role of ICAM-1 in the rapid MSC-mediated regulation of T cells, we knocked down ICAM-1 expression in MSCs using short interfering RNA (Supplementary Figure 11A). Consistent with the results obtained from the ICAM-1 blockade, ICAM-1 knockdown significantly weakened the inhibitory ability of MSCs (Supplementary Figures 11B–D), as evidenced by TCR signaling, Ca^2+^ level, and TNF-α and IFN-γ expression. Taken together, our results indicate that the rapid MSC-mediated suppression of activated T cells partially depends on ICAM-1-mediated cell-to-cell contact. In addition, we also found that recombinant ICAM-1 could reduce TCR signaling and Ca^2+^ levels, similar to MSCs (Supplementary Figure 12), suggesting that MSCs could also regulate T cells by secreting ICAM-1.

### MSCs Exert Rapid Immunosuppression on Activated T Cells Through CD43

**Figure 5:**
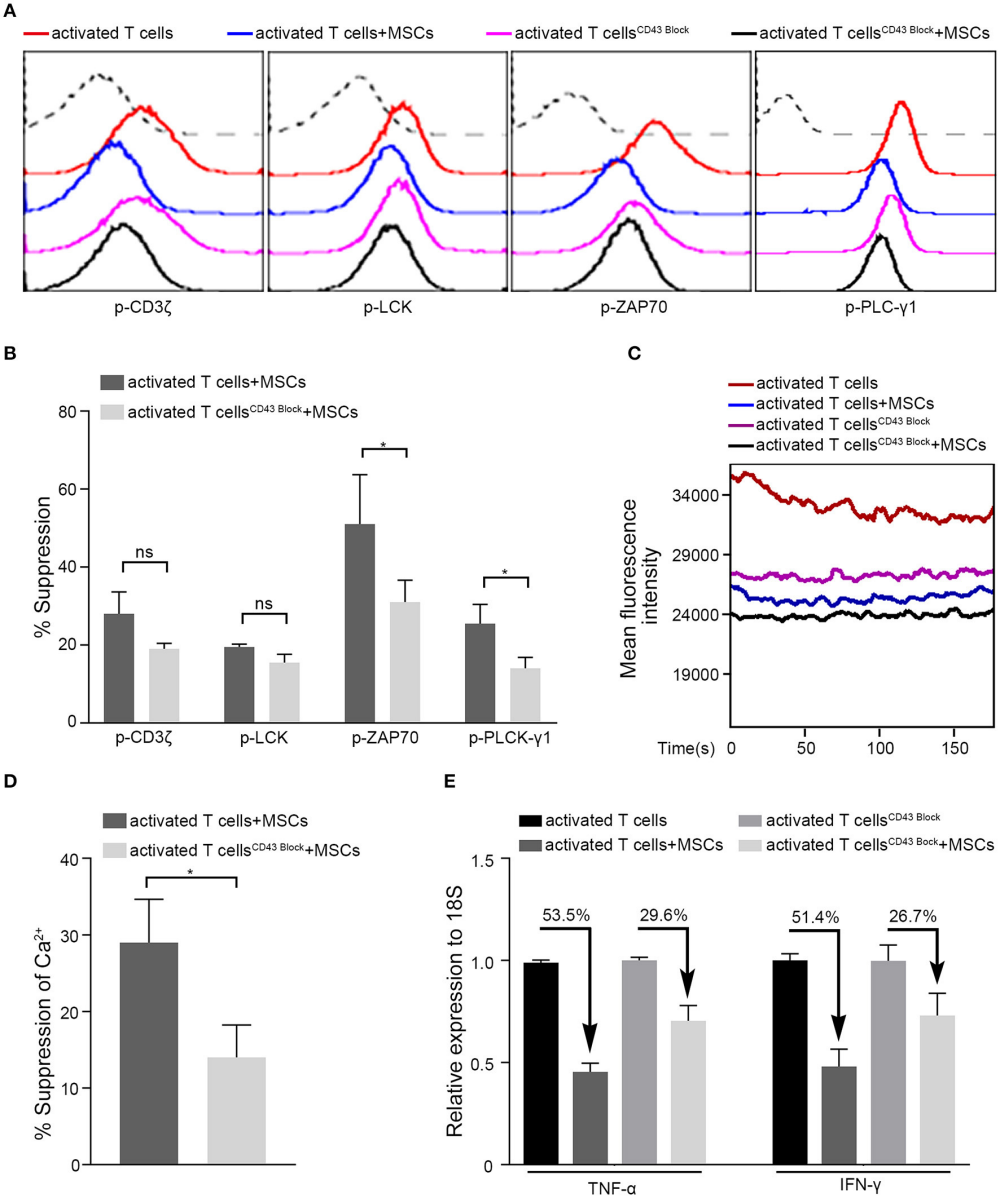
MSCs exert rapid immunosuppressive actions on T cells through CD43. Four groups were analyzed: activated T cells with and without MSCs coculture, CD43-blocked activated T cells with and without MSCs coculture. **(A)** The phosphorylation levels of CD3ζ, LCK, ZAP70, and PLC-γ1 in activated T cells of the four groups above, the dotted curve represents isotype. **(B)** Bar graphs show the suppression percentages of p-CD3ζ, p-LCK, p-ZAP70, and p-PLC-γ1. Comparison is between activated T cells cocultured with MSCs and CD43-blocked activated T cells cocultured with MSCs. **(C)** Cytosolic Ca^2+^ levels in activated T cells of the four groups were tested by flow cytometry. **(D)** The suppression percentages of the two MSCs-cocultured groups described in **(B)** are shown. **(E)** Quantitative reverse transcription–polymerase chain reaction (qRT-PCR) analysis of TNF-α and IFN-γ mRNA expressions in activated T cells from the four groups. The percentages of decrease after cocultured with MSCs are shown. All experiments were repeated three times. Data are presented as the mean ± SEM for each group. Significant differences are indicated as follows: ns, no statistical significance; *p < 0.05.

Having demonstrated that ICAM-1-mediated cell-to-cell contact plays a crucial role in the rapid immunoregulatory ability of MSCs, we next searched for the counterpart of ICAM1 on T cells. Among the known receptors of ICAM1 (51), LFA-1 (CD11a/CD18), MAC-1 (CD11b/CD18), and CD43 are the major receptors. MAC-1, a receptor found mainly on the surface of myeloid leukocytes, is reportedly expressed on <1% of T cells (52), whereas almost all T cells express LFA-1 and CD43 (53–55). Surprisingly, when we blocked the binding of LFA-1 with ICAM1 by introducing a small molecule inhibitor (RWJ50271) to the coculture system, we did not observe any significant change in the MSC-mediated regulation of activated T cells (Supplementary Figures 13A,B). However, blockade of CD43 impaired MSC-mediated suppression and restored the phosphorylation levels of the major TCR signaling-related kinases ZAP70 and PLCγ1 (Figures 5A,B). The ability of MSCs to downregulate Ca^2+^ levels was also diminished by the blockade of CD43 in our system (Figures 5C,D). In addition, after treatment with a CD43 blocking antibody, a deficiency in MSC-mediated immunosuppression of activated T cells was detected, as evidenced by the levels of TNF-α and IFN-γ transcription (Figure 5E). To confirm the role of CD43 in the rapid MSC-mediated regulation of activated T cells, we knocked down CD43 expression in Jurkat T cells using short interfering RNA (siRNA) (Supplementary Figure 13C). Consistent with the results obtained with CD43 neutralization, the inhibitory ability of MSCs was significantly diminished in CD43-knockdown Jurkat T cells (Supplementary Figures 13D–F), as evidenced by TCR signaling, Ca^2+^ level, and TNF-α and IFN-γ mRNA expression. These data indicate that MSCs might regulate TCR signaling and its downstream pathways via CD43-mediated negative signaling in activated T cells.

### MSC-Derived ICAM-1 Rapidly Alters CD43-Mediated TCR Microcluster Formation in Activated T Cells

**Figure 6:**
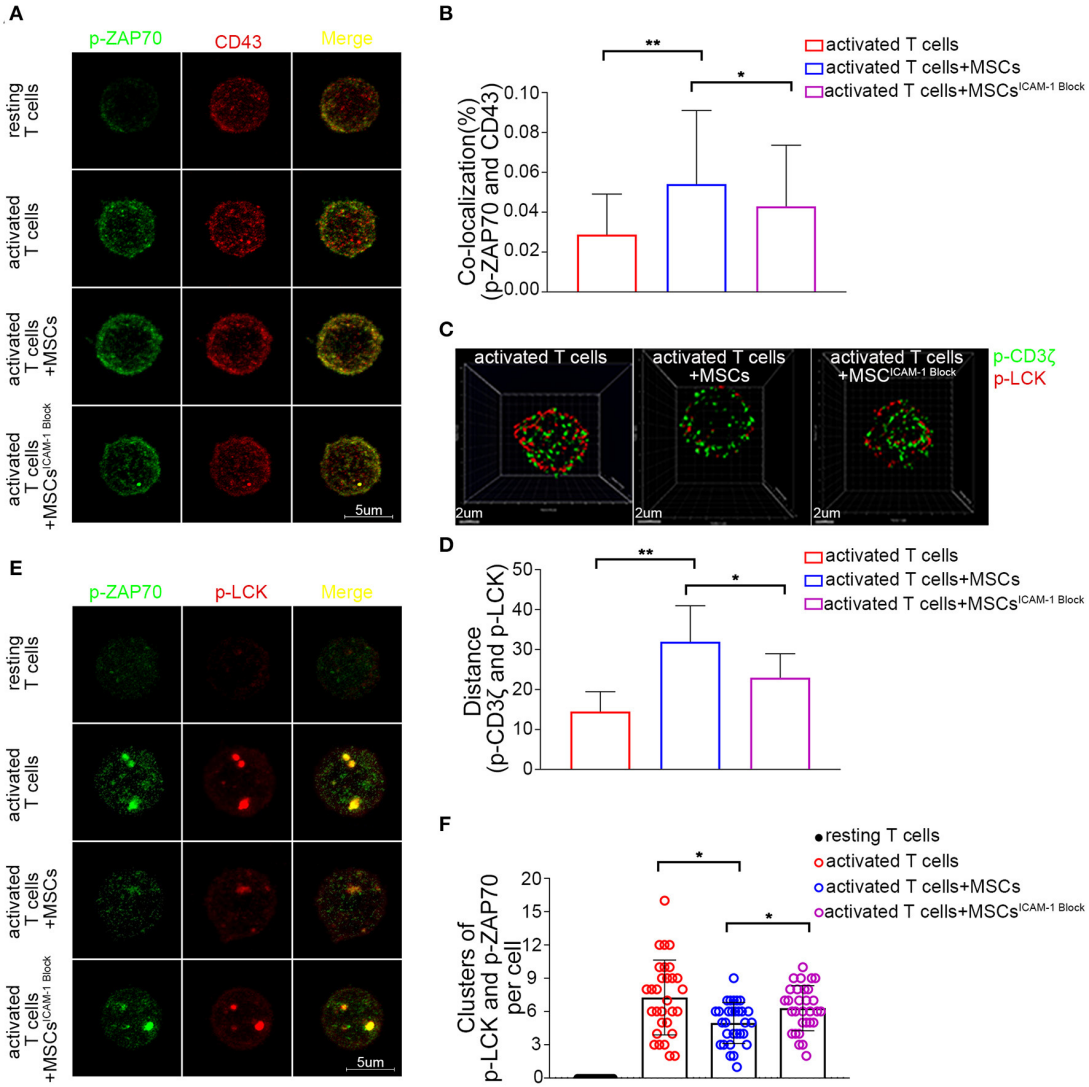
CD43-mediated TCR microcluster formation in activated T cells is rapidly altered by MSCs coculture through MSCs-derived ICAM-1. **(A)** Resting and activated T cells were plated on poly-lysine coated plates, and then fixed and stained for p-Zap70 (Y319) (green) and CD43 (red). Confocal images were collected. Far right shows overlay images. Four groups were analyzed: resting T cells, activated T cells, activated T cells cocultured with MSCs and activated T cells cocultured with ICAM-1-blocked MSCs. **(B)** The colocalization percentage of p-ZAP70 and CD43 in activated T cells, activated T cells cocultured with MSCs and activated T cells cocultured with ICAM-1-blocked MSCs. **(C)** Analog images display the distribution of p-CD3ζ (green) and p-LCK (red) in activated T cells. Three groups were analyzed: activated T cells, activated T cells cocultured with MSCs and activated T cells cocultured with ICAM-1-blocked MSCs. **(D)** Column shows the distances between p-CD3ζ and p-LCK in activated T cells of groups described in **(C)**. **(E)** Images show the distribution of microclusters of p-LCK (red) and p-ZAP70 (green) in resting and activated T cells, activated T cells cocultured with MSCs, and activated T cells cocultured with ICAM-1-blocked MSCs. Far right shows overlay images. **(F)** Numbers of formed p-ZAP70-containing microclusters per cell. Data are shown for the groups described in **(E)**. The data presented are representative of a minimum of three separate experiments. Panels are representative of at least 20 cells each. Significant differences are indicated as follows: *p < 0.05 and **p < 0.01.

Having found that the cross-linking of ICAM-1 and CD43 plays a crucial role in the rapid MSC-mediated modulation of activated T cells, we further focused on what was occurring inside the T cells. Once T cells are activated, many molecules are localized at the T-cell antigen–presenting cell interface, which is termed the immunological synapse (IS) (56). CD43 is the only molecule that is localized to the pole opposite the IS (57). Previous studies have found that the movement of CD43 may modulate T cell activation by sequestering proteins involved in regulating TCR signaling away from the site of TCR signaling (58, 59). In our system, we found that there was less colocalization of CD43 with p-LCK or p-ZAP70 in T cells after activation. Upon coculture with MSCs, there was no significant change in the colocalization of CD43 and p-LCK (data not shown); nevertheless, engagement of MSCs increased the percentage of p-ZAP70 that colocalized with CD43 (Figures 6A,B). Moreover, blocking ICAM-1 on MSCs rescued this MSC-mediated increase in the colocalization of CD43 and p-ZAP70 (Figures 6A,B). Activation of ZAP-70 represents a second critical checkpoint in T cell signaling, and binding of LCK to the phosphorylated Tyr319 (Y319) of ZAP-70, in turn, promotes the activation of LCK and further facilitates the activation of ZAP-70 through its phosphorylation at Tyr493 in the activation loop (60). Thus, we inspected the signaling components upstream of ZAP-70 and found that the colocalization of p-LCK and p-CD3ζ decreased in activated T cells cocultured with MSCs compared to that in monocultured T cells (Figure 6C). Moreover, the mean distance between p-LCK and p-CD3ζ was increased in activated T cells cocultured with MSCs (Figure 6D), and blockade of ICAM-1 in the cocultured MSCs partially restored both the colocalization of and the distance between p-LCK and p-CD3ζ (Figures 6C,D). These results suggest that MSCs may modulate TCR signaling by sequestering regulatory proteins away from the site of TCR signaling, possibly through their effects on CD43.

TCR clusters, which are formed in response to ligation of the TCR, act as a platform for the recruitment and activation of downstream effector molecules (61). Since our results suggested that MSCs could sequester signaling molecules, we further investigated whether they affected the formation of microclusters at immunological synapses. Using confocal microscopy, we observed that p-ZAP70 (Y319) and p-LCK (Y394) organized to form microclusters that accumulated within the membranes of activated T cells but not those of resting T cells (Figures 6E,F). However, the formation of microclusters (>0.8 μm in diameter) composed of p-Zap70 and p-LCK was decreased in the membranes of activated T cells cocultured with MSCs (Figures 6E,F). Moreover, blockade of ICAM1 impaired this MSC-mediated suppression of TCR microcluster formation (Figures 6E,F). These data suggest that MSC-derived ICAM1 may disrupt the macromolecular assembly of microclusters.

## Discussion

MSCs inhibit T cell activation, proliferation, and proinflammatory cytokine secretion (1, 2), and these actions have been shown to contribute to MSC therapy-based improvements in immune- and inflammation-mediated diseases (6, 7). The MSC-mediated inhibition of the T cell response depends largely on cell-to-cell contact and the release of soluble factors (5, 14). For instance, MSC-expressed indoleamine 2,3-dioxygenase (IDO) results in tryptophan depletion that inhibits allogeneic T cell proliferation (62), and MSCs directly inhibit the proliferation of alloreactive CD4+ and CD8+ T cells via galectin-1 (63). However, these MSC-mediated effects on T cells have typically been observed over days, weeks, or even months in both *in vitro* and *in vivo* studies. Recently, MSCs have shown great promise as preclinical and clinical therapies for severe acute conditions, such as septic shock and acute lung injury. Interestingly, such cases appear to involve early (within hours) and transient dampening of proinflammatory cytokines (64). This finding suggests that MSCs may possess a rapid immunomodulatory capacity that has not yet been fully elucidated. Here, using a T cell/MSC coculture system, we found that MSCs could downregulate TNF-α and IFN-γ mRNA in activated T cells within a short timeframe. We further observed that MSCs rapidly suppressed TCR signaling and its downstream pathways. Collectively, our present findings indicate that MSCs possess an unusual capacity for the rapid immunomodulation of activated T cells, which might facilitate their ability to control severe acute T cell-mediated disorders.

TCR signaling is critical for T cell immunity, as it activates the transcription factors that are required for T cell proliferation, cytokine secretion, and differentiation for effector function (43). Although MSCs were previously reported to suppress T-cell activation, inhibit T-cell proliferation, and reduce T-cell secretion of proinflammatory cytokines (16), no previous study has explored whether MSCs can regulate TCR signaling. Here, we demonstrate for the first time that MSCs may modulate TCR signaling, as evidenced by the altered phosphorylation of CD3ζ, LCK, ZAP-70, and PLC-γ1 (key kinases and phosphatases of T cell signaling) in activated T cells cocultured with MSCs. This finding laid the foundation for our comprehensive and in-depth exploration of MSC-mediated T cell regulation. Our results further showed that MSCs regulate Ca^2+^ signaling, which is of paramount importance in immunity. Regulated increases in cytosolic and organellar Ca^2+^ concentrations in lymphocytes have been shown to control complex and crucial effector functions (e.g., metabolism, proliferation, differentiation, antibody secretion, cytokine secretion, and cytotoxicity), and altered Ca^2+^ regulation in lymphocytes leads to various autoimmune, inflammatory, and immunodeficiency syndromes (39). Considering that MSCs regulate Ca^2+^ signaling, it is conceivable that they might have a multifaceted impact on T cells and could thus serve as the basis for promising therapeutic approaches in treating Ca^2+^-mediated disorders.

Previous studies have demonstrated that adhesion molecules are involved in the interactions between early T cells and bone marrow mesenchymal stromal cells (31). When MSCs were cocultured with activated T cells, ICAM-1 expression was increased (24), which upregulated the adhesion capability of T cells (31). ICAM-1 is a member of the immunoglobulin superfamily of adhesion molecules; these molecules contribute to cell-to-cell contact and cell-to-matrix interactions and are considered to be immune-promoting molecules (15). ICAM-1 and its receptors are critically involved in various inflammatory pathological diseases, such as experimental allergic encephalomyelitis, rheumatoid arthritis, and GVHD (65–67). Blockade of ligands or receptors has shown some beneficial effects in controlling these diseases in animal models. For example, MSC-derived ICAM-1 was reported to be critical for the MSC-mediated immunosuppression of T cells (24), and ICAM-1-modified MSCs remarkably alleviated inflammatory damage in mice with inflammatory bowel disease (49). Our results further revealed that ICAM-1 contributes to the rapid MSC-mediated suppression of TNF-α and IFN-γ in activated T cells and that, importantly, functional blockade of ICAM-1 significantly reversed the immunosuppressive effects of MSCs *in vitro*. These findings emphasize the important immunomodulatory role of ICAM-1 and may contribute to the future development of ICAM-1-based therapies for immune- and inflammation-mediated diseases.

Studies have shown the adhesive specificity of activated T cells and the important roles played by adhesion molecules in MSC-mediated immunosuppression (24, 31), but the exact mechanism of this suppression has not previously been determined. Here, we show that the rapid suppression of activated T cells by MSCs largely depends on cell-to-cell contact. We also confirmed the role of ICAM-1 in the rapid MSC-mediated suppression of activated T cells. Two main receptors of ICAM-1 are reported to be expressed on T lymphocytes (52–55). Here, we show that CD43 is most likely to be involved in the MSC-mediated suppression of activated T cells, as its blockade significantly reversed the rapid MSC-mediated suppression of TCR signaling. CD43 was previously found to modulate T cell activation via its selective exclusion from the T cell antigen–presenting cell contact site during the formation of T cell immune synapses (58). This exclusion of CD43 relies on its interaction with members of the ezrin-radixin-moesin (ERM) family of cytoskeletal adaptor proteins (68, 69). If the binding of CD43 to ERM is blocked, then CD43 fails to properly localize, resulting in inadequate activation of T cells. Ezrin phosphorylation has previously been shown to be essential for the recruitment of the major signaling kinase ZAP-70 to the IS (immunological synapse) (70). Interestingly, we found that the levels of phosphorylated ezrin and moesin were impaired in activated T cells following coculture with MSCs (Supplementary Figure 14), which might lead to the inaccurate localization of CD43. Based on our findings, it seems likely that ERM proteins participate in the rapid MSC-mediated suppression of T cells. Future work is needed to clarify the exact interaction between the TCR-CD43-ERM axis, as well as the subtleties of the underlying mechanisms.

In conclusion, we explored the molecular processes that occur in T cells whose activation is suppressed by direct interaction with MSCs. Our data reveal for the first time that MSCs trigger immediate Ca^2+^ signaling blockade in activated T cells by inhibiting TCR signaling through the ICAM-1/CD43 interaction, thereby suppressing the transcriptional expression of TNF-α and IFN-γ. These results shed new light on the immunomodulatory mechanism of MSCs used to treat aGVHD and other severe acute T cell-related diseases.

## Data Availability Statement

The raw data supporting the conclusions of this article will be made available by the authors, without undue reservation.

## Author Contributions

SZ, KH, and WX designed the experiments, performed the research, interpreted the data, and wrote the manuscript. KH collected the peripheral blood samples. XZ, JS, QL, JC, and TW participated in performing the research. XC and AX conceptualized and designed the study. XC supervised the research, interpreted the data, and wrote the manuscript. All authors contributed to the article and approved the submitted version.

## Conflict of Interest

The authors declare that the research was conducted in the absence of any commercial or financial relationships that could be construed as a potential conflict of interest.

## Abbreviations

aGVHD: acute graft-versus-host disease
CD: cluster of differentiation
GVHD: graft-versus-host disease
ICAM-1: intercellular adhesion molecule 1
IFN-γ: Interferon-γ
LCK: lymphocyte-specific protein tyrosine kinase
LFA-1: Lymphocyte function-associated antigen 1
MSCs: mesenchymal stromal cells
PBMC: peripheral blood mononuclear cell
PCR: polymerase chain reaction
PLC-γ1: phospholipase Cγ1
TCR: T cell receptor
TNF-α: Tumor Necrosis Factor-α
ZAP70: Zeta Chain of T Cell Receptor Associated Protein Kinase 70.

## References

[1] Le Blanc K Davies LC. Mesenchymal stromal cells and the innate immune response. Immunol Lett. (2015) 168:140–6. 10.1016/j.imlet.2015.05.004 25982165

[2] Chiossone L Conte R Spaggiari GM Serra M Romei C Bellora F. Mesenchymal stromal cells induce peculiar alternatively activated macrophages capable of dampening both innate and adaptive immune responses. Stem Cells. (2016) 34:1909–21. 10.1002/stem.2369 27015881

[3] Cao W Cao K Cao J Wang Y Shi Y. Mesenchymal stem cells and adaptive immune responses. Immunol Lett. (2015) 168:147–53. 10.1016/j.imlet.2015.06.003 26073566

[4] Han Y Li X Zhang Y Han Y Chang F Ding J. Mesenchymal stem cells for regenerative medicine. Cells. (2019) 8:886. 10.3390/cells8080886 PMC6721852 31412678

[5] Nauta AJ Fibbe WE. Immunomodulatory properties of mesenchymal stromal cells. Blood. (2007) 110:3499–506. 10.1182/blood-2007-02-069716 17664353

[6] Wang Y Chen X Cao W Shi Y. Plasticity of mesenchymal stem cells in immunomodulation: pathological and therapeutic implications. Nat Immunol. (2014) 15:1009–16. 10.1038/ni.3002 25329189

[7] Wang M Yuan Q Xie L. Mesenchymal stem cell-based immunomodulation: properties and clinical application. Stem Cells Int. (2018) 2018:3057624. 10.1155/2018/3057624 30013600 PMC6022321

[8] Le Blanc K Rasmusson I Sundberg B Gotherstrom C Hassan M Uzunel M. Treatment of severe acute graft-versus-host disease with third party haploidentical mesenchymal stem cells. Lancet. (2004) 363:1439–41. 10.1016/S0140-6736(04)16104-7 15121408

[9] Le Blanc K Frassoni F Ball L Locatelli F Roelofs H Lewis I. Mesenchymal stem cells for treatment of steroid-resistant, severe, acute graft-versus-host disease: a phase II study. Lancet. (2008) 371:1579–86. 10.1016/S0140-6736(08)60690-X 18468541

[10] Reinisch A Etchart N Thomas D Hofmann NA Fruehwirth M Sinha S. Epigenetic and in vivo comparison of diverse MSC sources reveals an endochondral signature for human hematopoietic niche formation. Blood. (2015) 125:249–60. 10.1182/blood-2014-04-572255 25406351 PMC4287636

[11] Sima Y Chen Y. MSC-based therapy in female pelvic floor disorders. Cell Biosci. (2020) 10:104. 10.1186/s13578-020-00466-4 32944218 PMC7488254

[12] Behnke J Kremer S Shahzad T Chao CM Böttcher-Friebertshäuser E Morty RE. MSC based therapies-new perspectives for the injured lung. J Clin Med. (2020) 9:682. 10.3390/jcm9030682 32138309 PMC7141210

[13] FrançoisMRomieu-MourezRLiMGalipeauJ. Human MSC suppression correlates with cytokine induction of indoleamine 2,3-dioxygenase and bystander M2 macrophage differentiation. Mol Ther. (2012) 20:187–95. 10.1038/mt.2011.18921934657

[14] Davies LC Heldring N Kadri N Le Blanc K. Mesenchymal stromal cell secretion of programmed death-1 ligands regulates t cell mediated immunosuppression. Stem Cells. (2017) 35:766–76. 10.1002/stem.2509 27671847 PMC5599995

[15] Ren G Roberts AI Shi Y. Adhesion molecules: key players in mesenchymal stem cell-mediated immunosuppression. Cell Adh Migr. (2011) 5:20–2. 10.4161/cam.5.1.13491 20935502 PMC3038091

[16] Duffy MM Ritter T Ceredig R Griffin MD. Mesenchymal stem cell effects on T-cell effector pathways. Stem Cell Res Ther. (2011) 2:34. 10.1186/scrt75 21861858 PMC3219065

[17] Masterson CH Curley GF Laffey JG. Modulating the distribution and fate of exogenously delivered MSCs to enhance therapeutic potential: knowns and unknowns. Intensive Care Med Exp. (2019) 7:41. 10.1186/s40635-019-0235-4 31346794 PMC6658643

[18] Liu S Liu F Zhou Y Jin B Sun Q Guo S. Immunosuppressive property of MSCs mediated by cell surface receptors. Front Immunol. (2020) 11:1076. 10.3389/fimmu.2020.01076 32849489 PMC7399134

[19] Panes J Garcia-Olmo D Van Assche G Colombel JF Reinisch W Baumgart DC. Expanded allogeneic adipose-derived mesenchymal stem cells (Cx601) for complex perianal fistulas in Crohn's disease: a phase 3 randomised, double-blind controlled trial. Lancet. (2016) 388:1281–90. 10.1016/S0140-6736(16)31203-X 27477896

[20] Panes J Garcia-Olmo D Van Assche G Colombel JF Reinisch W Baumgart DC. Long-term efficacy and safety of stem cell therapy (Cx601) for complex perianal fistulas in patients with Crohn's disease. Gastroenterology. (2018) 154:1334–42.e4. 10.1053/j.gastro.2017.12.020 29277560

[21] Duijvestein M Vos AC Roelofs H Wildenberg ME Wendrich BB Verspaget HW. Autologous bone marrow-derived mesenchymal stromal cell treatment for refractory luminal Crohn's disease: results of a phase I study. Gut. (2010) 59:1662–9. 10.1136/gut.2010.215152 20921206

[22] Dhere T Copland I Garcia M Chiang KY Chinnadurai R Prasad M. The safety of autologous and metabolically fit bone marrow mesenchymal stromal cells in medically refractory Crohn's disease - a phase 1 trial with three doses. Aliment Pharmacol Ther. (2016) 44:471–81. 10.1111/apt.13717 27385373

[23] Barda-Saad M Rozenszajn LA Ashush H Shav-Tal Y Ben Nun A Zipori D. Adhesion molecules involved in the interactions between early T cells and mesenchymal bone marrow stromal cells. Exp Hematol. (1999) 27:834–44. 10.1016/S0301-472x(99)00010-7 10340399

[24] Ren G Zhao X Zhang L Zhang J L'Huillier A Ling W. Inflammatory cytokine-induced intercellular adhesion molecule-1 and vascular cell adhesion molecule-1 in mesenchymal stem cells are critical for immunosuppression. J Immunol. (2010) 184:2321–8. 10.4049/jimmunol.0902023 20130212 PMC2881946

[25] Silva-Carvalho AE Rodrigues LP Schiavinato JL Alborghetti MR Bettarello G Simoes BP. GVHD-derived plasma as a priming strategy of mesenchymal stem cells. Stem Cell Res Ther. (2020) 11:156. 10.1186/s13287-020-01659-x 32299501 PMC7164240

[26] Peng Y Chen X Liu Q Zhang X Huang K Liu L. Mesenchymal stromal cells infusions improve refractory chronic graft versus host disease through an increase of CD5+ regulatory B cells producing interleukin 10. Leukemia. (2015) 29:636–46. 10.1038/leu.2014.225 25034146

[27] Sadick JS Boutin ME Hoffman-Kim D Darling EM. Protein characterization of intracellular target-sorted, formalin-fixed cell subpopulations. Sci Rep. (2016) 6:33999. 10.1038/srep33999 27666089 PMC5036045

[28] Ziprin P Alkhamesi NA Ridgway PF Peck DH Darzi AW. Tumour-expressed CD43 (sialophorin) mediates tumourmesothelial cell adhesion. Biol Chem. (2004) 385:755–61. 10.1515/BC.2004.092 15449712

[29] Pennino D Eyerich K Scarponi C Carbone T Eyerich S Nasorri F. IL-17 amplifies human contact hypersensitivity by licensing hapten nonspecific Th1 cells to kill autologous keratinocytes. J Immunol. (2010) 184:4880–8. 10.4049/jimmunol.0901767 20357258

[30] Cossarizza A Chang HD Radbruch A Acs A Adam D Adam-Klages S. Guidelines for the use of flow cytometry and cell sorting in immunological studies (second edition). Eur J Immunol. (2019) 49:1457–973. 10.1002/eji.201970107 31633216 PMC7350392

[31] Suva D Passweg J Arnaudeau S Hoffmeyer P Kindler V. *In vitro* activated human T lymphocytes very efficiently attach to allogenic multipotent mesenchymal stromal cells and transmigrate under them. J Cell Physiol. (2008) 214:588–94. 10.1002/jcp.21244 17786951

[32] Ferrara JL Reddy P. Pathophysiology of graft-versus-host disease. Semin Hematol. (2006) 43:3–10. 10.1053/j.seminhematol.2005.09.001 16412784

[33] Sun Y Tawara I Toubai T Reddy P. Pathophysiology of acute graft-versus-host disease: recent advances. Transl Res. (2007) 150:197–214. 10.1016/j.trsl.2007.06.003 17900507 PMC2084257

[34] Elgaz S Kuci Z Kuci S Bonig H Bader P. Clinical use of mesenchymal stromal cells in the treatment of acute graft-versus-host disease. Trans Med Hemotherapy. (2019) 46:27–34. 10.1159/000496809 31244579 PMC6558336

[35] Zhao L Chen S Yang P Cao H Li L. The role of mesenchymal stem cells in hematopoietic stem cell transplantation: prevention and treatment of graft-versus-host disease. Stem Cell Res Ther. (2019) 10:182. 10.1186/s13287-019-1287-9 31227011 PMC6588914

[36] Bonig H Kuci Z Kuci S Bakhtiar S Basu O Bug G. Children and adults with refractory acute graft-versus-host disease respond to treatment with the mesenchymal stromal cell preparation “MSC-FFM”-outcome report of 92 patients. Cells. (2019) 8:1577. 10.3390/cells8121577 31817480 PMC6952775

[37] Trebak M Kinet JP. Calcium signalling in T cells. Nat Rev Immunol. (2019) 19:154–69. 10.1038/s41577-018-0110-7 30622345 PMC6788797

[38] Lewis RS. Calcium signaling mechanisms in T lymphocytes. Annu Rev Immunol. (2001) 19:497–521. 10.1146/annurev.immunol.19.1.497 11244045

[39] Randriamampita C Trautmann A. Ca^2+^ signals and T lymphocytes; “new mechanisms and functions in Ca^2+^ signalling”. Biol Cell. (2004) 96:69–78. 10.1016/j.biolcel.2003.10.008 15093129

[40] Stocker N Dulery R Battipaglia G Brissot E Mediavilla C Sestili S. Impact of cyclosporine A concentration on acute graft-vs.-host disease incidence after haploidentical hematopoietic cell transplantation. Eur J Haematol. (2019) 103:10–17. 10.1111/ejh.13233 30958904

[41] Xhaard A Launay M Sicre de Fontbrune F Michonneau D Sutra Del Galy A Coman T. A monocentric study of steroid-refractory acute graft-versus-host disease treatment with tacrolimus and mTOR inhibitor. Bone Marrow Transplant. (2020) 55:86–92. 10.1038/s41409-019-0633-y 31413313

[42] Huang B Lin X Zhang Z Zhang Y Zheng Z Zhong C. Comparison of tacrolimus and cyclosporine combined with methotrexate for graft versus host disease prophylaxis after allogeneic hematopoietic cell transplantation. Transplantation. (2020) 104:428–36. 10.1097/TP.0000000000002836 31283681

[43] Smith-Garvin JE Koretzky GA Jordan MS. T cell activation. Annu Rev Immunol. (2009) 27:591–619. 10.1146/annurev.immunol.021908.132706 19132916 PMC2740335

[44] Courtney AH Lo WL Weiss A. TCR signaling: mechanisms of initiation and propagation. Trends Biochem Sci. (2018) 43:108–23. 10.1016/j.tibs.2017.11.008 29269020 PMC5801066

[45] Gaud G Lesourne R Love PE. Regulatory mechanisms in T cell receptor signalling. Nat Rev Immunol. (2018) 18:485–97. 10.1038/s41577-018-0020-8 29789755

[46] Rasmusson I Ringdén O Sundberg B Le Blanc K. Mesenchymal stem cells inhibit lymphocyte proliferation by mitogens and alloantigens by different mechanisms. Exp Cell Res. (2005) 305:33–41. 10.1016/j.yexcr.2004.12.013 15777785

[47] Najar M Raicevic G Fayyad-Kazan H De Bruyn C Bron D Toungouz M. Impact of different mesenchymal stromal cell types on T-cell activation, proliferation and migration. Int Immunopharmacol. (2013) 15:693–702. 10.1016/j.intimp.2013.02.020 23499510

[48] Najar M Raicevic G Fayyad-Kazan H De Bruyn C Bron D Toungouz M. Immune-related antigens, surface molecules and regulatory factors in human-derived mesenchymal stromal cells: the expression and impact of inflammatory priming. Stem Cell Rev Rep. (2012) 8:1188–98. 10.1007/s12015-012-9408-1 22983809

[49] Li X Wang Q Ding L Wang YX Zhao ZD Mao N. Intercellular adhesion molecule-1 enhances the therapeutic effects of MSCs in a dextran sulfate sodium-induced colitis models by promoting MSCs homing to murine colons and spleens. Stem Cell Res Ther. (2019) 10:267. 10.1186/s13287-019-1384-9 31443680 PMC6708236

[50] Tang B Li X Liu Y Chen X Li X Chu Y. The therapeutic effect of ICAM-1-overexpressing mesenchymal stem cells on acute graft-versus-host disease. Cell Physiol Biochem. (2018) 46:2624–35. 10.1159/000489689 29763906

[51] Meyer DM Dustin ML Carron CP. Characterization of intercellular adhesion molecule-1 ectodomain (sICAM-1) as an inhibitor of lymphocyte function-associated molecule-1 interaction with ICAM-1. J Immunol. (1995) 155:3578–84. 7561056

[52] Wedi B Elsner J Czech W Butterfield JH Kapp A. Modulation of intercellular adhesion molecule 1 (ICAM-1) expression on the human mast-cell line (HMC)-1 by inflammatory mediators. Allergy. (1996) 51:676–84. 8904994

[53] Christensen JE Andreasen SO Christensen JP Thomsen AR. CD11b expression as a marker to distinguish between recently activated effector CD8(+) T cells and memory cells. Int Immunol. (2001) 13:593–600. 10.1093/intimm/13.4.593 11282998

[54] Walling BL Kim M. LFA-1 in T cell migration and differentiation. Front Immunol. (2018) 9:952. 10.3389/fimmu.2018.00952 29774029 PMC5943560

[55] Rosenstein Y Park JK Hahn WC Rosen FS Bierer BE Burakoff SJ. CD43, a molecule defective in Wiskott-Aldrich syndrome, binds ICAM-1. Nature. (1991) 354:233–5. 10.1038/354233a0 1683685

[56] Alarcon B Mestre D Martinez-Martin N. The immunological synapse: a cause or consequence of T-cell receptor triggering? Immunology. (2011) 133:420–5. 10.1111/j.1365-2567.2011.03458.x 21631496 PMC3143353

[57] Delon J Kaibuchi K Germain RN. Exclusion of CD43 from the immunological synapse is mediated by phosphorylation-regulated relocation of the cytoskeletal adaptor moesin. Immunity. (2001) 15:691–701. 10.1016/S1074-7613(01)00231-X 11728332

[58] Sperling AI Sedy JR Manjunath N Kupfer A Ardman B Burkhardt JK. TCR signaling induces selective exclusion of CD43 from the T cell-antigen-presenting cell contact site. J Immunol. (1998) 161:6459–62. 9862667

[59] Manjunath N Correa M Ardman M Ardman B. Negative regulation of T-cell adhesion and activation by CD43. Nature. (1995) 377:535–8. 10.1038/377535a0 7566153

[60] Thill PA Weiss A Chakraborty AK. Phosphorylation of a tyrosine residue on zap70 by Lck and its subsequent binding via an SH2 domain may be a key gatekeeper of T cell receptor signaling *in vivo*. Mol Cell Biol. (2016) 36:2396–402. 10.1128/Mcb.00165-16 27354065 PMC5007795

[61] Purbhoo MA Liu HB Oddos S Owen DM Neil MAA Pageon SV. Dynamics of subsynaptic vesicles and surface microclusters at the immunological synapse. Sci. Signal. (2010) 3:ra36. 10.1126/scisignal.2000645 20460647

[62] Meisel R Zibert A Laryea M Gobel U Daubener W Dilloo D. Human bone marrow stromal cells inhibit allogeneic T-cell responses by indoleamine 2,3-dioxygenase-mediated tryptophan degradation. Blood. (2004) 103:4619–21. 10.1182/blood-2003-11-3909 15001472

[63] Gieseke F Bohringer J Bussolari R Dominici M Handgretinger R Muller I. Human multipotent mesenchymal stromal cells use galectin-1 to inhibit immune effector cells. Blood. (2010) 116:3770–9. 10.1182/blood-2010-02-270777 20644118

[64] Schlosser K Wang JP Dos Santos C Walley KR Marshall J Fergusson DA. Effects of mesenchymal stem cell treatment on systemic cytokine levels in a phase 1 dose escalation safety trial of septic shock patients. Crit Care Med. (2019) 47:918–25. 10.1097/CCM.0000000000003657 30720538 PMC6629173

[65] Haghayegh Jahromi N Marchetti L Moalli F Duc D Basso C Tardent H. Intercellular adhesion molecule-1 (ICAM-1) and ICAM-2 differentially contribute to peripheral activation and CNS entry of autoaggressive Th1 and Th17 cells in experimental autoimmune encephalomyelitis. Front Immunol. (2019) 10:3056. 10.3389/fimmu.2019.03056 31993059 PMC6970977

[66] Lavigne P Benderdour M Shi Q Lajeunesse D Fernandes JC. Involvement of ICAM-1 in bone metabolism: a potential target in the treatment of bone diseases? Expert Opin Biol Ther. (2005) 5:313–20. 10.1517/14712598.5.3.313 15833069

[67] Holler E Ertl B Hintermeier-Knabe R Roncarolo MG Eissner G Mayer F. Inflammatory reactions induced by pretransplant conditioning–an alternative target for modulation of acute GvHD and complications following allogeneic bone marrow transplantation? Leuk Lymphoma. (1997) 25:217–24. 9168432 10.3109/10428199709114161

[68] Allenspach EJ Cullinan P Tong J Tang Q Tesciuba AG Cannon JL. ERM-dependent movement of CD43 defines a novel protein complex distal to the immunological synapse. Immunity. (2001) 15:739–50. 10.1016/s1074-7613(01)00224-2 11728336

[69] Shaffer MH Dupree RS Zhu P Saotome I Schmidt RF McClatchey AI. Ezrin and moesin function together to promote T cell activation. J Immunol. (2009) 182:1021–32. 10.4049/jimmunol.182.2.1021 19124745 PMC3491660

[70] Ilani T Khanna C Zhou M Veenstra TD Bretscher A. Immune synapse formation requires ZAP-70 recruitment by ezrin and CD43 removal by moesin. J Cell Biol. (2007) 179:733–46. 10.1083/jcb.200707199 18025306 PMC2080902

