# Supplementary material for: Mesenchymal Stromal Cells Rapidly Suppress TCR Signaling-Mediated Cytokine Transcription in Activated T Cells Through the ICAM-1/CD43 Interaction
Source: Front Immunol. 2021 Feb 22;12:609544. doi: 10.3389/fimmu.2021.609544 (PMC7937648; doi:10.3389/fimmu.2021.609544)
Supplement: Supplementary file 1 [file Data_Sheet_1.pdf]

Supplementary Materials

Figures

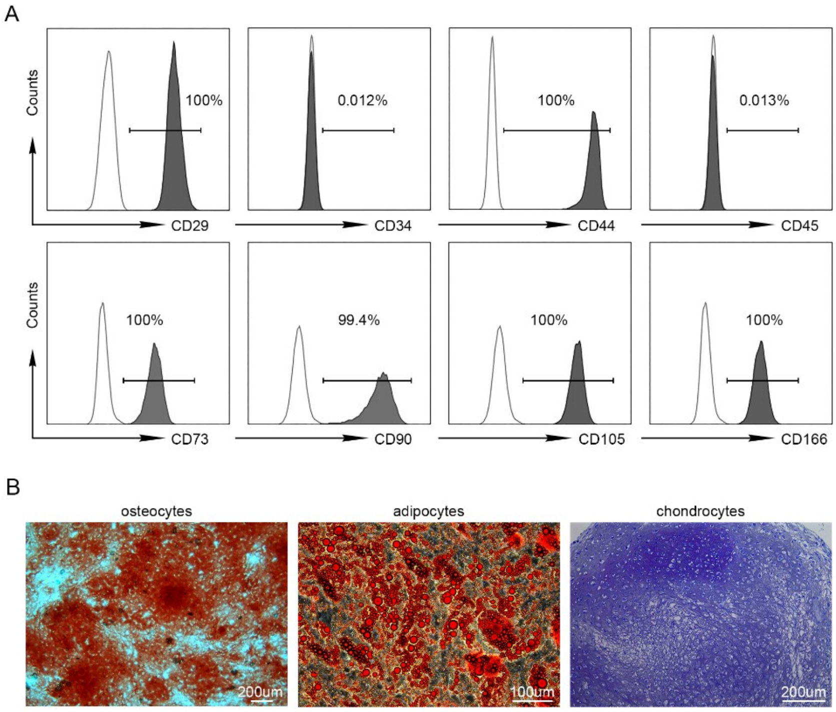

Figure S1. The characteristics of MSCs in vitro.

(A) The MSCs surface expression levels of CD29, CD44, CD73, CD90, CD105, CD166, CD34, and CD45 were analyzed by flow cytometry.

(B) Oil red O, Alizarin red S, and Toluidine blue O staining were used to assess the adipogenic, osteogenic, and chondrogenic differentiations of MSCs cultured under conditions that favored their differentiation capacity into these lineages. Scale bar are marked in the figures.

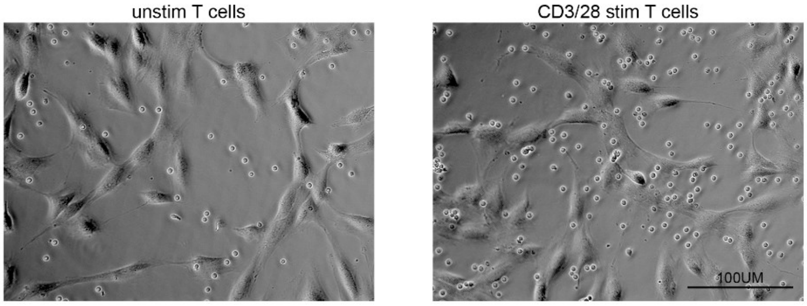

Figure S2. Adhesion ability of T cells to MSCs before and after coculture with MSCs for 1 hour.

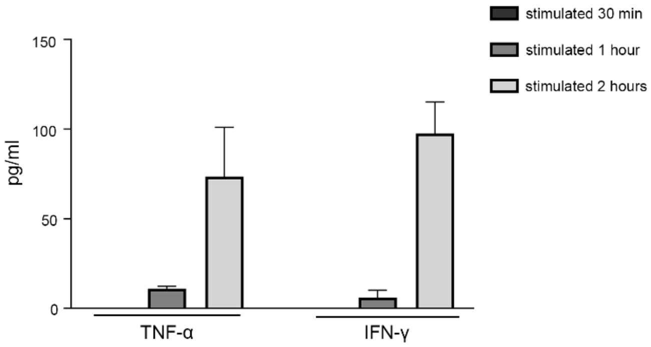

Figure S3. IFN-γ and TNF-α protein levels in supernatants of stimulated T cells. Via ELISA, IFN-γ and TNF-α secretion were evaluated after activation for 30 min,1 h, 2 h.

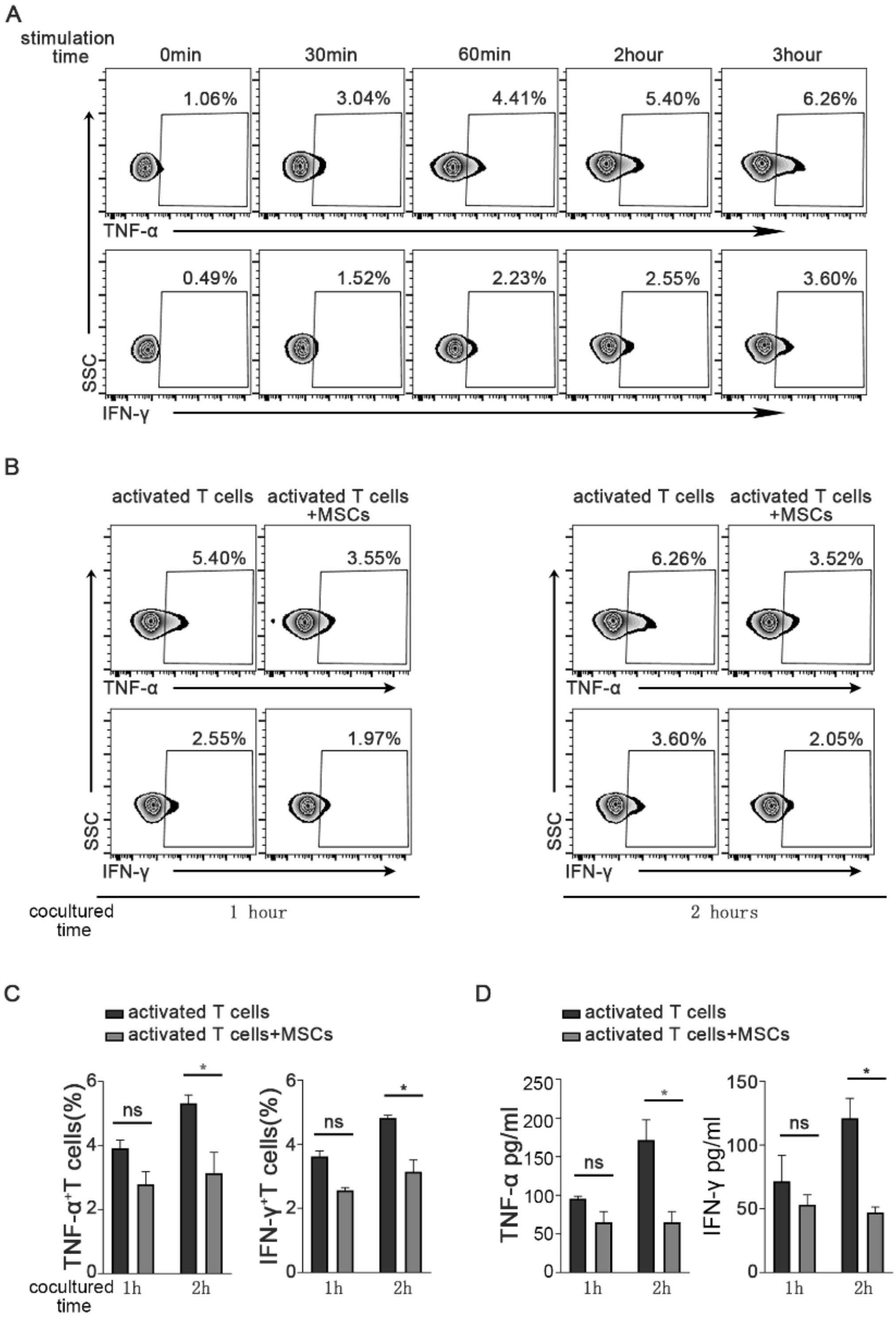

A
stimulation time
0min
30min
60min
2hour
3hour
1.06%
3.04%
4.41%
5.40%
6.26%
TNF-α
0.49%
1.52%
2.23%
2.55%
3.60%
SSC
IFN-γ
B
activated T cells
activated T cells +MSCs
5.40%
3.55%
6.26%
3.52%
TNF-α
2.55%
1.97%
3.60%
2.05%
SSC
IFN-γ
cocultured time
1 hour
2 hours
C
activated T cells
activated T cells+MSCs
TNF-α+T cells(%)
IFN-γ+T cells(%)
ns
*
cocultured time
1h
2h
D
activated T cells
activated T cells+MSCs
TNF-α pg/ml
IFN-γ pg/ml
ns
*
1h
2h

Figure S4 TNF-α and IFN-γ protein levels in activated T cells and their alternations after MSCs coculture. Activated T cells: T cells were stimulated by CD3 mAb and CD28 mAb for 1 h.

(A) TNF-α and IFN-γ protein staining of activated T cells were evaluated via flow cytometry before and after stimulation by CD3 mAb and CD28 mAb for 30min, 1 h, 2 h, 3 h.

(B) TNF-α and IFN-γ protein levels in activated T cells cocultured with or without MSCs for 1 h, 2 h.

(C) Statistical graph of TNF-α and IFN-γ protein levels in activated T cells cocultured with or without MSCs for 1 h, 2 h in (B).rrrrr

(D) TNF-α and IFN-γ secreted by activated T cells cocultured with or without MSCs were analyzed by ELISA at 1 h, 2 h.

All presented data were obtained from three repeated experiment. Significant differences are indicated as follows: ns: no statistical significance, *$p<0.05$.

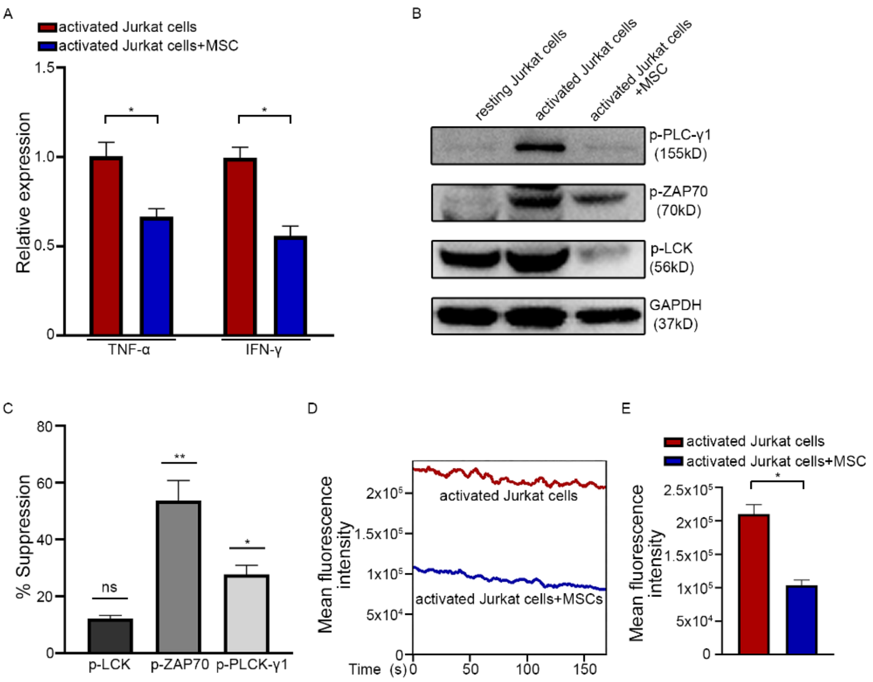

Figure S5. MSCs exert rapid immunosuppression on Jurkat cells the same way as on T cells. Jurkat were treated as described for T cells in Figure 1E.

(A) The mRNA expressions of TNF-α and IFN-γ in activated Jurkat cells were evaluated before and after coculture with MSCs.

(B) The phosphorylation of CD3ζ, ZAP70, and PLC-γ1 in activated Jurkat cells were analyzed by Western blotting.

(C) The phosphorylation of p-LCK, p-ZAP70, and p-PLC-γ1 in activated Jurkat cells were tested by flow cytometry, statistical graph of suppression percentage of phosphorylation levels was shown.

(D) Calcium levels in activated Jurkat cells were tested via flow cytometry.

(E) Statistical graph of the mean fluorescence intensity in (D) was shown.

All experiments were repeated three times. Significant differences are indicated as follows: ns: no statistical significance, *p < 0.05 and **p < 0.01.

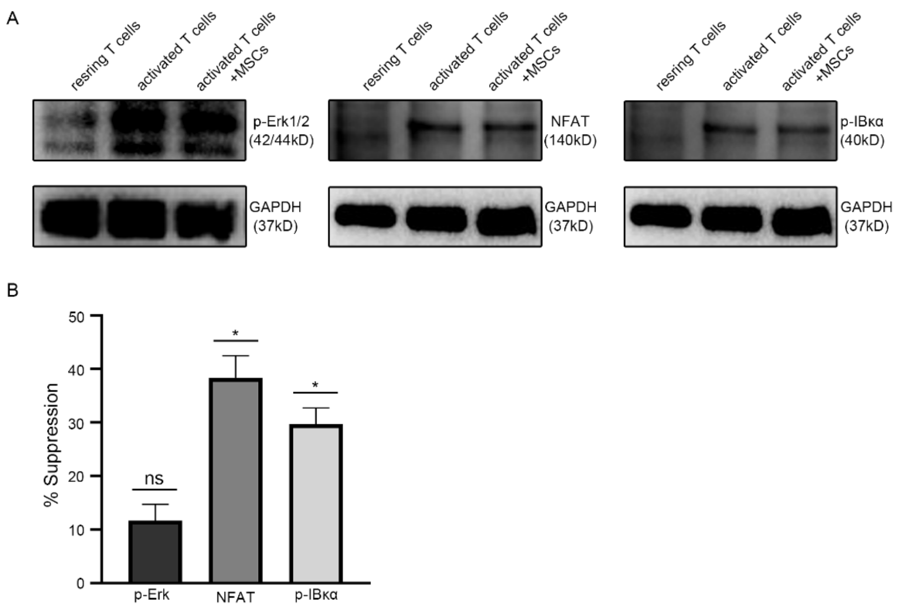

Figure S6. NFAT signaling, NF-κB signaling, and ERK signaling in activated T cells and their changes after cocultured with MSCs.

(A) p-ERK, NFAT, and p-IBκα expression in activated T cells were detected by western blot before and after MSCs coculture.

(B) Statistical graph of p-ERK, NFAT, and p-IBκα expression in activated T cells in (A), the percentage of suppression by MSCs is shown.

All experiments were repeated three times. Significant differences are indicated as follows: ns: no statistical significance, *p < 0.05.

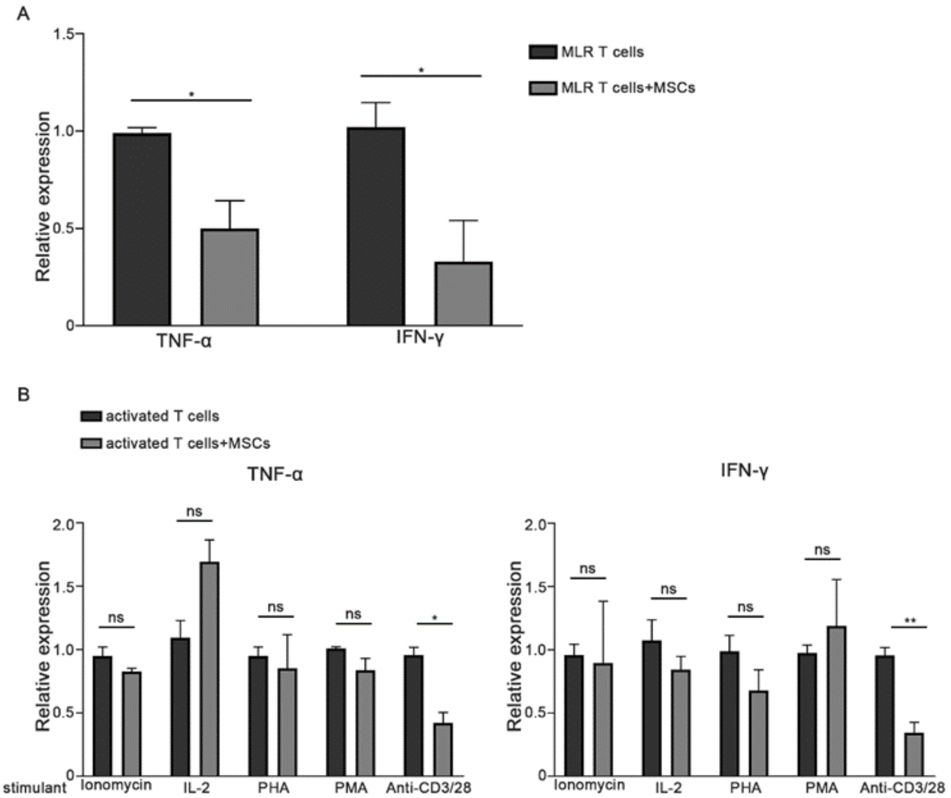

Figure S7. mRNA expressions of TNF-α and IFN-γ in T cells after stimulation by different stimuli before and after MSCs coculture.

(A) The mRNA levels of TNF-α and IFN-γ in T cell after MLR (mixed lymphocyte reaction) and their changes after MSCs coculture.

(B) The mRNA expression of TNF-α and IFN-γ in T cells stimulated by IL-2 and mitogens as Ionomycin, PHA and PMA, and their changes after MSCs coculture, compared with anti-CD3/CD28.

All presented data were obtained from three repeated experiment. Significant differences are indicated as follows: ns: no statistical significance, *$p < 0.05$,**$p < 0.01$.

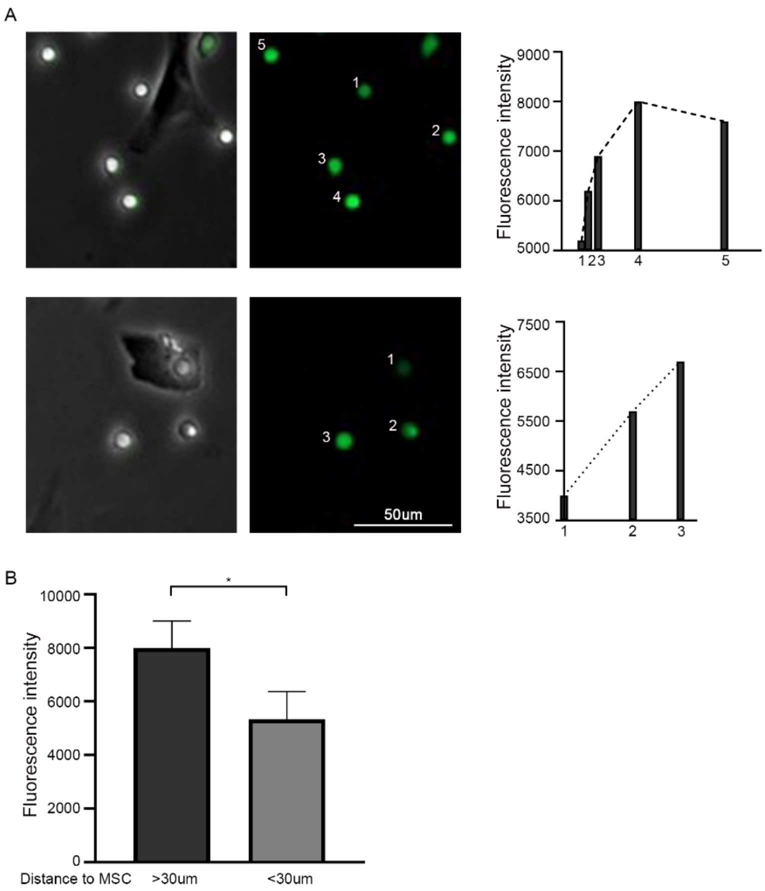

Figure S8. Calcium levels of activated T cells were related to their proximity to MSCs.

(A) Images displayed the Ca^{2+} levels of activated T cells (the small round cells) cocultured with or without MSCs (the large spindle-shaped cells), Ca^{2+} levels were expressed as fluorescence intensity. Column shows the fluorescence intensities of the cells bearing the corresponding numbers in the pictures.

(B) The fluorescence intensities of T cells located >30 μm and <30 μm relative to the nearest MSC.

All experiments were repeated three times. Significant differences are indicated as $*p < 0.05$.

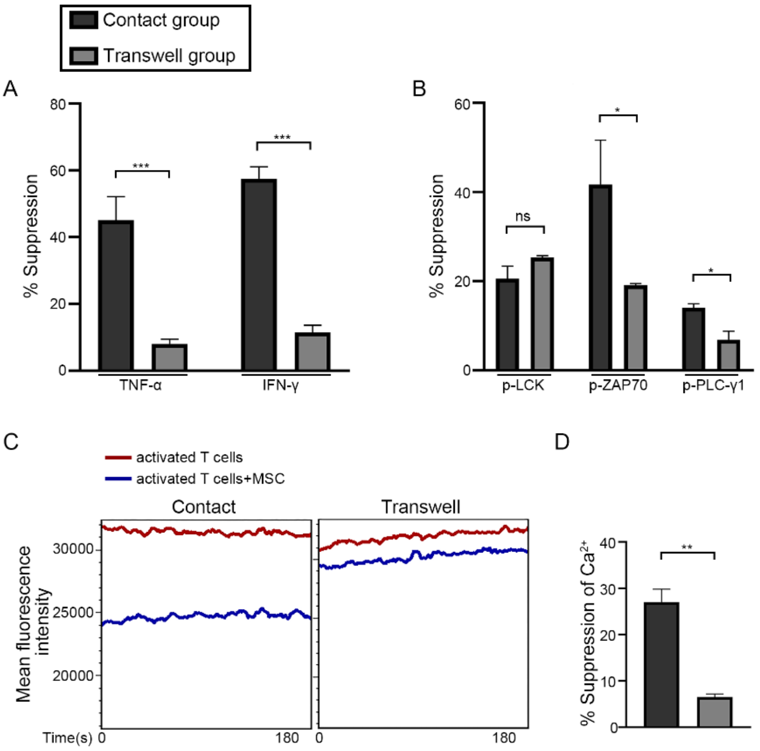

Figure S9. Cell-to-cell contact played a critical role in the rapid immunomodulatory capacity of MSCs.

(A) The mRNA levels of TNF-α and IFN-γ in activated T cells were detected before and after MSC coculture. Percentage of suppression of contact and Transwell group is shown.

(B) The phosphorylation of p-LCK, p-ZAP70, and p-PLC-γ1 in activated T cells were tested by flow cytometry. The suppression percentage of phosphorylation is shown.

(C) Calcium levels in activated T cells were evaluated by flow cytometry. The suppression percentage of the average fluorescence intensity is shown.

(D) Statistical graph of Calcium levels in activated T cells in (C), bar graphs show the percentages of suppression by MSCs.

Significant differences are indicated as follows: ns: no statistical significance, *$p < 0.05$, **$p < 0.01$, and ***$p < 0.001$.

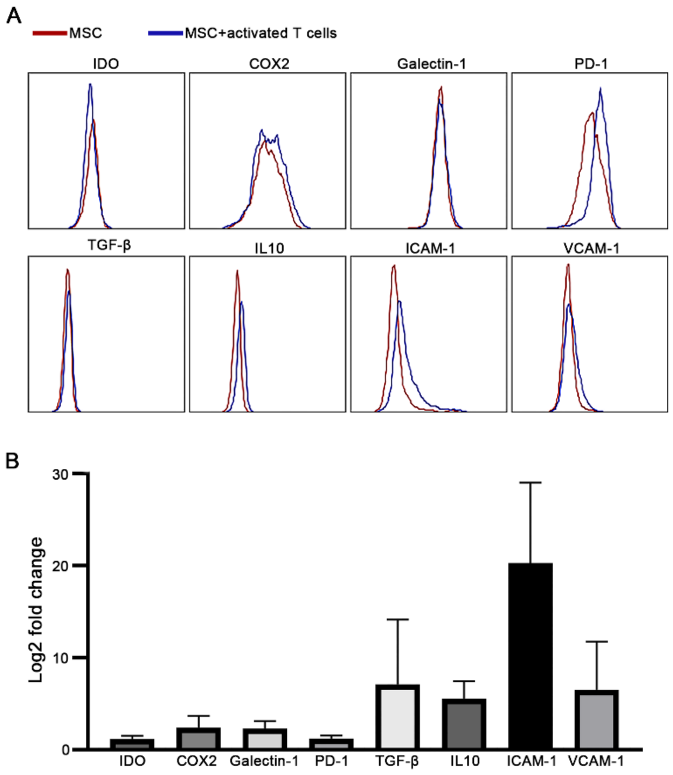

Figure.S10 Assessment of molecules that are associated with the immunomodulatory properties of MSCs

The expression changes of immunosuppression-associated molecules of MSCs after cocultured with activated T cells.

(A) Molecules associated with the **immunomodulatory capacity of MSCs** were evaluated by flow cytometry.

(B) mRNA expression levels of immunosuppression-associated molecules in MSCs after cocultured with activated T cells for 1 hour.

All experiments were repeated at least three times using the sixth-passage MSCs. Data

are presented as the mean ± SEM for each group.

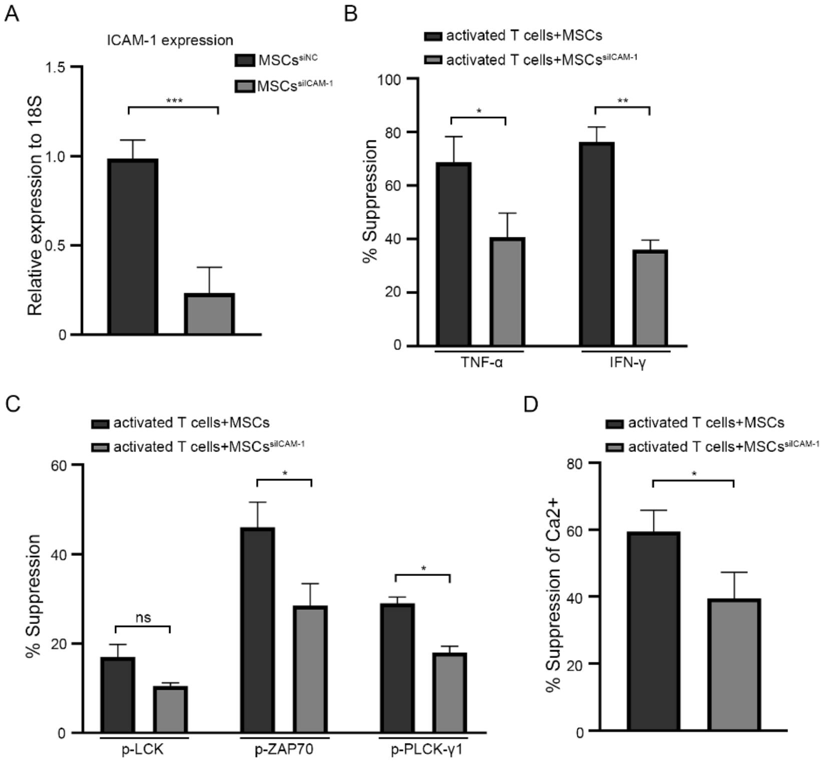

Figure S11. ICAM-1 siRNA transfection had a similar effect to that of ICAM-1 blocking antibodies on MSC-mediated T cell suppression.

Three different treated groups were compared for the following experiments: activated T cells, activated T cells cocultured with MSCs, activated T cells cocultured with $MSCs^{siICAM\text{-}1}$.

(A) Interference effects of ICAM-1 siRNA applied to MSCs.

(B) Quantitative reverse transcription–polymerase chain reaction (qRT-PCR) analysis of the mRNA abundances of TNF-α and IFN-γ in activated T cells, shown as the percentage of suppression.

(C) Phosphorylation levels of the three major proteins LCK, ZAP70, PLC-γ1 in activated T cells were evaluated by flow cytometry. The percentage of suppression is shown.

(D) Cytosolic calcium levels in activated T cells were examined via flow cytometry. The suppression percentage is shown.

All presented data were obtained from three repeated experiment. Significant differences are indicated as follows: ns: no statistical significance, $*p < 0.05$, and $**p < 0.01$.

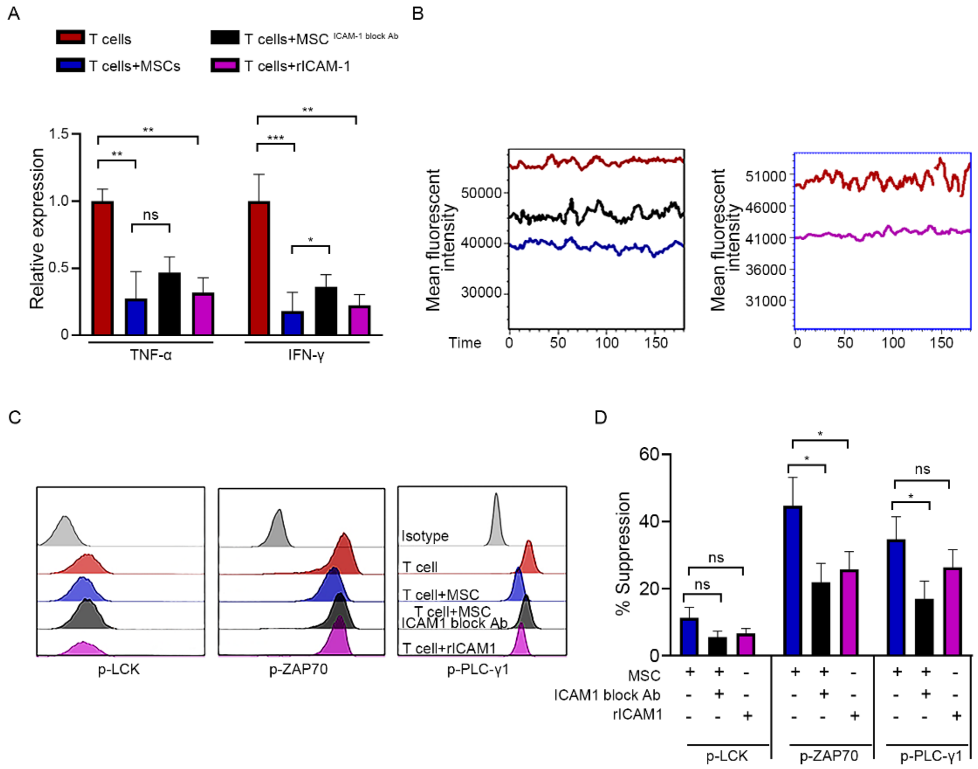

Figure S12. ICAM-1 recombinant protein (rICAM-1) has a similar effect as MSC-mediated rapid modulation on the T cell

Four different treated groups were compared for the following experiments: activated T cells, activated T cells cocultured with MSCs, activated T cells cocultured with MSCs^{siICAM1}, activated T cells cocultured with rICAM-1 protein.

(A) Quantitative reverse transcription–polymerase chain reaction (qRT-PCR) analysisof the mRNA abundances of TNF-α and IFN-γ in activated T cells, shown as the relative expression to 18S.

(B) The intracellular calcium level of T cells was tested by flow cytometry.

(C) Phosphorylation levels of the three major proteins LCK, ZAP70, PLC-γ1 were

tested by flow cytometry.

(D) The percentage of suppression of picture C is shown.

All presented data were obtained from three repeated experiment. Significant differences are indicated as follows: ns: no statistical significance, *p < 0.05, **p < 0.01 and***p < 0.001.

.

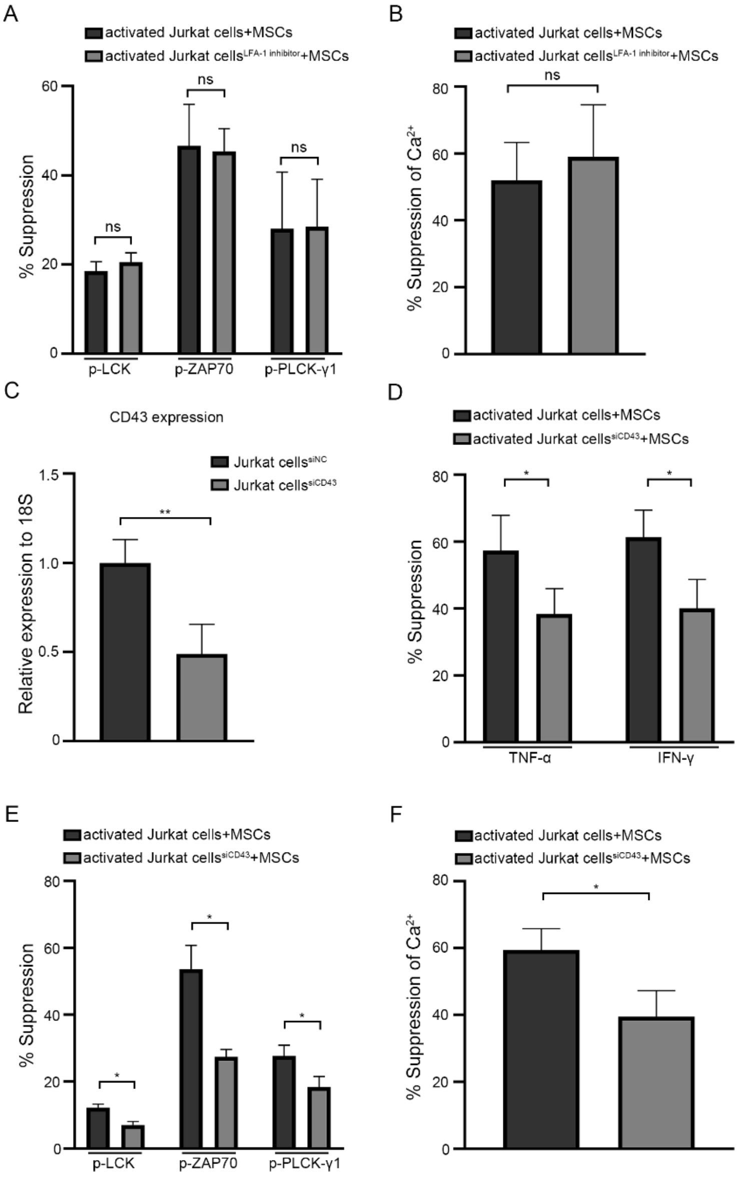

Figure S13 siRNA-mediated CD43 knockdown in Jurkat cells had effects similar to those of CD43 blocking antibodies in T cells cocultured with MSCs.

Jurkat cells were treated as described for PBMC T cells in Figure 1E.

(A) Phosphorylation of the three major proteins LCK, ZAP70, PLC-γ1 were tested by flow cytometry. The four different treated groups were assessed, included activated T cells, activated T cells cocultured with MSCs, activated T cells treated with inhibitor of LFA-1/ICAM-1, activated T cells cocultured with MSCs after treated with inhibitor of

LFA-1/ICAM-1. The percentage of suppression is shown.

(B) Intracellular calcium level was tested by flow cytometry of the four groups described in A. The percentage of suppression is shown.

(C) siRNA-mediated knockdown of CD43 in Jurkat cells.

(D) Quantitative reverse transcription–polymerase chain reaction (qRT-PCR) analysis of the mRNA abundances of TNF-α and IFN-γ in activated T cells. Four different treated groups were assessed, as activated T cells, activated T cells cocultured with MSCs, activated T^{siCD43} cells, activated T^{siCD43} cells cocultured with MSCs. The percentage of suppression is shown.

(E) Phosphorylation of the three major proteins LCK, ZAP70, PLC-γ1 were tested by flow cytometry. The percentage of suppression is shown.

(F) Intracellular calcium level was tested by flow cytometry. The percentage of suppression is shown.

The presented data were from three repeated experiments. Significant differences are indicated as follows: ns: no statistical significance, $^{*}p < 0.05$. and $^{**}p < 0.01$.

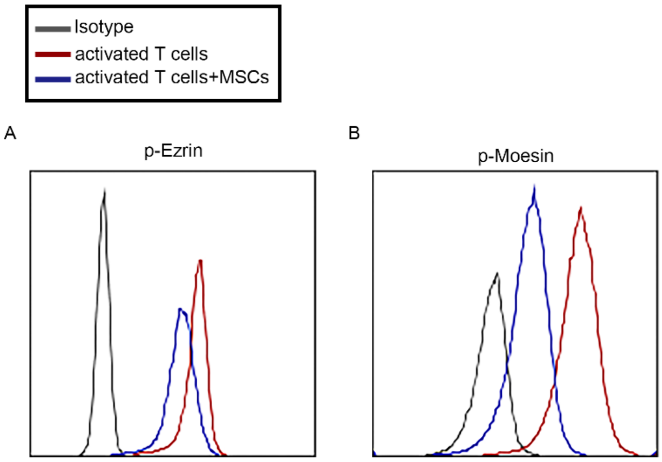

Figure S14. The phosphorylation levels of ezrin and moesin in T cells are abrogated within 1 hour of MSC coculture.

Tables

Table S1. Human Sequence of Specific Primers Used for q-PCR Analysis

Genes	Forward Sequence	Reverse Sequence
18S	CCCGAAGCGTTTACTTTGA	CAAATGCTTTCGCTCTGGT
TNF-α	GCTGCACTTTGGAGTGATCG	TCACTCGGGGTTCGAGAAGA
IFN-γ	TTCAGCTCTGCATCGTTTTGG	TCCGCTACATCTGAATGACCTG
CD43	TTGGTCTCTACTAGCGAGCC	TGGCTCAGGTAAAGGGGAAC
ICAM1	CAGGGAATATGCCCAAGCTA	TGAACCATGATTGCACCACT
VCAM1	AAGGTTCCTAGCGTGTACCC	CTGCCTTTGTTTGGGTTCGA
COX2	CTCCTGTGCCTGATGATTGC	AACTGATGCGTGAAGTGCTG
Galectin1	GGCAAAGACAGCAACAACCT	GCGGTTGGGGAACTTGAATT
TGF-β	CTGCAAGTGGACATCAACG	TCCTTGCGGAAGTCAATGT
IL-10	GTTGCCAAGCCTTGTCTGA	GGCCTTGCTCTTGTTTTCA
PD-L1	TGGCCATATTCTGGTGTCAA	CCAACACCACAAGGAGGAGT
IDO	GGCAAAGGTCATGGAGATGT	CTGCAGTCTCCATCACGAAA

Table S2. Human Primary Antibodies Used for Western blot

Antigen	Host	Source	Dilution	Clone/Cat
p-LCK（Y493）	Mouse	Abcam	1:500	ab201567
p-ZAP70（Y319）	Rabbit	Cell Signaling Technology	1:500	65E4
p-PLCγ1（Y783）	Rabbit	Cell Signaling Technology	1:500	2821
NFAT	Rabbit	Affinity	1:500	DF7189
p-ERK1/2 (Y202/Y204)	Rabbit	Cell Signaling Technology	1:1000	9101S
p-Ikα (S32)	Rabbit	Cell Signaling Technology	1:1000	2859S
GAPDH	Mouse	Cell Signaling Technology	1:2000	D4C6R

Table S3. Secondary Reagents Used for Western blot

	Source	Dilution
Anti-rabbit	Cell Signaling Technology	1:2000
Anti-mouse	Cell Signaling Technology	1:2000

Table S4. Human Primary Antibodies for Flow Cytometry

Antigen	Source	Dilution	Clone/Cat
CD56 (Pe-cy7)	Thermo Fisher	1:50	CMSSB
CD19 (PE)	BD biosciences	1:50	555413
CD14 (Percp-cy5.5)	Biolegend	1:50	63D3
CD3 (V450)	BD biosciences	1:50	UCHT1
CD43 (APC)	Thermo Fisher	1:50	L10
p-ZAP70(Y319) (APC)	Thermo Fisher	1:50	n3kobu5
p-LCK (Y493)	Abcam	1:50	ab201567
p-PLCγ1 (Y783)	Cell Signaling Technology	1:50	2821
p-Ezrin (Thr567)	Thermo Fisher	1:500	PA5-37763
p-Moesin (Thr558)	Thermo Fisher	1:500	PA5-38679
ICAM1(APC)	Thermo Fisher	1:50	HA58
VCAM1 (Pe-cy7)	Thermo Fisher	1:50	STA
TGFβ (PE)	R&D System	1:50	9016
IDO (Pe-cy7)	Thermo Fisher	1:50	eyedio
COX2 (PE)	Thermo Fisher	1:50	COX 229
Galectin	Thermo Fisher	1:50	JM13-37
IL10 (Pe-cy7)	Thermo Fisher	1:50	JES39D7
PD-L1 (APC)	BD biosciences	1:50	M1H1
F-actin (AF647)	Thermo Fisher	1:50	A22287
CD45RO (PE)	BD biosciences	1:20	UCHL1
CD197	BD biosciences	1:50	557648
CD8 (APC)	BD biosciences	1:20	RPA-T8
Anti-CD3 zeta (Y83)	Abcam	1:100	EP776(2)Y
CD29(APC)	Thermo Fisher	1:50	TS2/16
CD44(APC)	BD biosciences	1:50	G44-26
CD73(PE)	BD biosciences	1:50	AD2
CD90(APC)	BD biosciences	1:50	5E10
CD105(PE)	Thermo Fisher	1:20	MHCD10505
CD166(PE)	Thermo Fisher	1:50	3A6
CD34(FITC)	Thermo Fisher	1:50	4H11
CD45(Pe-cy7)	Thermo Fisher	1:50	HI30

Table S5. Secondary Reagents Used for Flow Cytometry

	Conjugate(s)	Source	Dilution
Anti-rabbit	Alexa Fluor 488	Thermo Fisher	1:1000
Anti-rabbit	Alexa Fluor 555	Thermo Fisher	1:1000
Anti-mouse	Alexa Fluor 488	Thermo Fisher	1:1000

Table S6. Human Primary Antibodies Used for Immunofluorescence Staining

Antigen	Host	Source	Dilution	Clone/Cat
CD43 (PE)	Mouse	Thermo Fisher	1:400	L10
p-LCK（Y493）	Mouse	Abcam	1:500	ab201567
p-ZAP70（Y319）	Rabbit	Cell Signaling Technology	1:400	65E4
p-Ezrin (Thr567)	Rabbit	Thermo Fisher	1:200	PA5-37763
p-Moesin (Thr558)	Rabbit	Thermo Fisher	1:200	PA5-38679
Anti-CD3zeta (Y83)	Rabbit	Abcam	1:200	EP776(2)Y

Table S7. Secondary Reagents Used for Immunofluorescence Staining

	Conjugate(s)	Source	Dilution
Anti-rabbit	Alexa Fluor 488	Thermo Fisher	1:500
Anti-rabbit	Alexa Fluor 555	Thermo Fisher	1:500
Anti-mouse	Alexa Fluor 640	Thermo Fisher	1:500
